# Norms for scaling up small and sick newborn care: an overview of reviews

**DOI:** 10.7189/jogh.15.04290

**Published:** 2025-11-21

**Authors:** Natalie A Strobel, Georgia Whisson, Derek Swe, Amy Budrikis, Karen M Edmond

**Affiliations:** 1 Edith Cowan University, Kurongkurl Katitjin, Perth, Australia; 2 World Health Organization, Newborn and Child Health and Development Unit, Geneva, Switzerland

## Abstract

**Background:**

The World Health Organization (WHO) and the United Nations Children’s Fund (UNICEF) currently have no benchmarks or ‘norms’ for scaling up small and sick newborn (SSN) service delivery in health facilities in low- and middle-income countries (LMICs). Our objective was to understand which systematic reviews had addressed the following norms in the last five years: number of SSN beds per live births in a district or similar administrative unit (admission beds); space requirements for SSN units, including mother-infant dyads (space); health workforce ratios in SSN units (workforce); and travel time to health facilities with SSN units (travel time).

**Methods:**

We searched for systematic reviews of admission beds, space, workforce and travel time norms for SSN under 28 days of age and their mothers in all health facilities and countries, regardless of infant gestational age and birth weight, that had been published in the previous five years (2018–23). For beds, space, and workforce norms, we searched for reviews of prevalence, incidence, mean and median estimates. For the travel time norm, we searched for reviews of estimates of effect, *i.e.* dichotomous (*e.g*. relative risks) or continuous measures (*e.g.* mean differences).

**Results:**

We identified 9110 records and included eight systematic reviews published in the last five years: two related to space, five to workforce, and one to travel time norms. We found no reviews for admission bed norms. Two reviews included high income countries only, while three included tertiary neonatal intensive care units only. The reviews provided estimates of mean space requirements in SSN units, health workforce ratios of doctors and nurses, and optimal travel time to health facilities for SSN. Seven of the eight reviews had high risk of bias.

**Conclusions:**

Despite the high burden of SSN in LMICs and the need to scale up hospital care, there have been few systematic reviews into this topic, and rigorous syntheses of evidence are lacking. The WHO and the UNICEF have now commissioned four systematic reviews. The next steps will be to analyse real-world country-level data and develop implementation guidance.

**Registration:**

PROSPERO: CRD42023417847, CRD42023451302, CRD42023478512, CRD42023453644.

Small and sick newborn (SSN) care is defined as care for the infant who is born preterm (<37 weeks), with low birth weight (<2.5kg), or who becomes sick with medical or surgical conditions throughout the first 28 days of life [1]. The global Every Newborn Action Plan (ENAP) defines three levels of newborn care [2] (**Box 1**): level 1 (primary care clinics), level 2 (district hospitals or equivalent), and level 3 (tertiary or specialist hospitals). The fourth global ENAP target (target 4) is to have at least one level 2 SSN care unit in every district (or equivalent) in every country [1]. However, there are large variations in population size, birth rates, and admission policies across districts and countries. For example, a typical district (*woreda*) in Ethiopia has about 200 000 inhabitants, while the population of an average district in India can range from one to two million persons. Population density, terrain, and the distance that families have to travel to reach healthcare also vary widely across countries [3].

Box 1 **Definitions used in the study**

**Newborn care**

SSN: infant who is born preterm (<37 weeks), low birth weight (<2.5kg) or who becomes sick with medical or surgical conditions throughout the first 28 days of life.

SSN unit: unit providing level 2 care for a SSN. This includes 12-, 20-, and 24-bedded units; and wards or rooms for newborns or mother-infant dyads. Other terms commonly used include special care nursery, special care neonatal unit, special newborn care unit, district hospital nursery, level 2 unit, and comprehensive EmOC neonatal unit.

Episode of neonatal severe morbidity: episode that would necessitate care in a SSN unit, *e.g.* prematurity (<32, <34, <37 weeks), serious infections (sepsis, pneumonia, meningitis), birth asphyxia (including meconium aspiration, encephalopathy), birth trauma, and common problems such as respiratory distress syndrome, seizures, hypoglycaemia, hyperbilirubinaemia, hypo/hyperthermia, and birth defects in infants under 28 days.

Neonate: infant aged from birth to <28 days.

Admission: infant who has been admitted into a bed in a hospital (excludes normal deliveries, and infants who are well and born after a normal delivery).

EmONC: a set of life-saving interventions that treat the major obstetric and newborn causes of morbidity and mortality. To assess the level of care, these functions are classified as basic or comprehensive EmONC levels of care. BEmONC services comprise: administration of parenteral antibiotics to prevent puerperal infection or treat abortion complications; administration of parenteral anticonvulsants for treatment of eclampsia and preeclampsia; administration of parenteral uterotonic drugs for postpartum haemorrhage; manual removal of the placenta; assisted vaginal delivery (vacuum extractions); removal of retained products of conception. Neonatal resuscitation. If a facility has provided all seven key basic EmONC functions in the last three months, it is considered a basic EmONC facility. Comprehensive EmONC services comprise caesarean sections and blood transfusions, in addition to basic EmONC functions.

**Levels of health care provision**

Primary (level 1) essential newborn care: immediate newborn care (thorough drying, skin to skin contact of the newborn with the mother, delayed cord clamping, hygienic cord care); neonatal resuscitation for those who need it), early initiation and support for exclusive breastfeeding, routine care (vitamin K, eye care and vaccinations, weighing and clinical examinations); prevention of mother to child transmission of HIV, assessment management and referral of bacterial infections, jaundice and diarrhoea, feeding problems, birth defects and other problems, pre discharge advice on mother and baby care and follow up.

Secondary (level 2) special newborn care – additional to level 1: comfort and pain management, kangaroo mother care, assisted feeding for optimal nutrition (cup feeding and nasogastric feeding), safe administration of oxygen, prevention of apnoea, detection and management of neonatal infection, detection and management of hypoglycaemia, jaundice, anaemia, and neonatal encephalopathy, seizure management, safe administration of intravenous fluids, detection and referral management of birth defects. Transition to intensive care: continuous positive airway pressure, exchange transfusion, detection and management of necrotising enterocolitis, specialised follow up of infants at high risk (including preterm).

Tertiary (level 3) intensive newborn care – additional to level 1 and 2: advanced feeding support (*e.g.* parenteral nutrition), mechanical/assisted ventilation including intubation, screening and treatment for retinopathy of prematurity, surfactant treatment, investigation and management of birth defects, paediatric surgery, genetic services.

Much work has been done to establish the norms for availability of emergency obstetric care (EmOC) services, including delineating basic and comprehensive EmOC facilities per population (**Box 1**) [4,5]. Comprehensive EmOC facilities provide caesarean sections and blood transfusion, and are considered to be similar to traditional district hospitals and level 2 facilities [4,5]. Current recommendations call for at least five EmOC facilities (four basic EmOC and one comprehensive EmOC facility) per 500 000 population, as well as that EmOC facilities should be no more than two hours travel time from a family’s home. These recommendations were formulated in 1987 based on expert opinion and are currently being reviewed under a process called ‘emergency obstetric and newborn care re-visioning’ [4,5]. However, there are currently no benchmarks or ‘norms’ for scaling up SSN service delivery in healthcare facilities in low-and middle-income countries (LMICs), especially level 2 facilities.

Norms for scaling up of health care interventions should incorporate measures of both quality of care (including structure, processes, and outcomes of care) [6] and access to care (including availability, accessibility, affordability, and acceptability) [7,8]. At a meeting held in 2022, experts from a World Health Organization (WHO) technical advisory group recommended that SSN norms should initially focus on three structural norms (admission beds, space in SSN units, health workforce) and one access norm (travel time to health facilities). They therefore framed four questions (**Box 2**) focussed on the number of SSN beds per live births in a district or similar administrative unit (admission beds); space requirements for SSN units, including mother-infant dyads (space); health workforce ratios in SSN units (workforce); and travel time to health facilities with SSN units (travel time).

Box 2 **Research questions used in the study**

**Structural norms**

Determining the number of beds needed for SSN care in a district or equivalent administrative unit:

*What are the hospital admission incidence rates and length of hospital stay for SSN aged under 28 days?*

Determining space requirements for SSN units:

*What is the space needed in SSN units including for kangaroo mother care (KMC) and mother-infant dyads?*

Determining the health care workforce in SSN units:

*What are the optimal health workforce ratios in SSN care units?*

**Access norms**

Determining the optimal distribution of SSN units in a population (district or equivalent administrative unit):

*Is the time taken to travel to a health facility associated with perinatal or neonatal mortality?*

We thus designed this study to identify any recent systematic reviews that had addressed these four questions and to assess their methodological quality.

## METHODS

After registering the study protocols on PROSPERO (CRD42023417847, CRD42023451302, CRD42023478512, CRD42023453644), we used standardised methods outlined in the Cochrane Handbook for Overview of Reviews to search for relevant systematic reviews [9]. We followed the PRIOR guidance in reporting our findings [10] (Figure S1 in the **Online Supplementary Document**).

### Search strategy

We searched MEDLINE (*via* Ovid), Embase (*via* Ovid), the Cochrane Library, Epistemonikos, and PROSPERO for reviews looking at structure (beds, space, workforce) and access (travel time) norms (Figures S2–5 and Tables S1–4 in the **Online Supplementary Document**). We ran the searches in March 2023 for beds, August 2023 for space, November 2023 for workforce, and July 2023 for travel time, limiting our queries to the last five years (2018 to date of search), as we sought to investigate the most recent evidence to inform the development of WHO guidelines. There were no restrictions on language or stage of the review (*e.g.* protocol, in progress, in press, fully published). We used Covidence systematic review software to manage all stages [11].

### Screening criteria, selection process, and data extraction

We developed our criteria (**Box 2**) separately for structural norms (beds, space, health workforce) and access norms (travel time).

For structural norms, we included systematic reviews or meta-analyses of studies of point or period prevalence, incidence rates, ratios or continuous measures such as means and medians of any estimates. Their study population had to be neonates under 28 days of age and caregiver-infant dyads in all health facilities (health centres and secondary and tertiary level hospitals, including specialised hospitals), regardless of gestational age and birth weight (*i.e.* we considered preterm and small for gestational age infants). In terms of interventions, we included all reviews and meta-analyses focussing on beds, space and health workforce that might provide any relevant estimates. We excluded case series, abstracts, and editorial/opinion articles.

For access norms, we included systematic reviews or meta-analyses of randomised controlled trials (RCTs), non-randomised studies of interventions, cohort or case-control studies with comparator groups, provided they focussed on neonates under 28 days of age and caregiver-infant dyads in all health facilities (health centres and secondary and tertiary level hospitals including specialised hospitals), regardless of gestational age and birth weight (*i.e.* we considered preterm and small for gestational age infants). Regarding the intervention/exposure criterion, we considered all studies of geographic access comparing different travel times, and excluded those that only included distance in terms of kilometres or mileage or other forms of access. For outcomes, the studies included in the reviews had to assess association with perinatal or neonatal mortality outcomes – *i.e.* stillbirths or early or late neonatal deaths (all-cause or cause-specific mortality). We excluded case series, abstracts, and editorial/opinion articles.

At least two review authors (GW, DS, AB, NS, KE) independently screened titles and abstracts and assessed the full texts of all identified systematic reviews for eligibility. Disagreements were resolved by a third author. We then generated and pretested a data extraction form, after which two review authors (GW, DS) independently extracted the following data from each review: study designs, country, setting, populations, interventions, and outcomes (**Table 1**, **Table 2**). We resolved discrepancies through discussion they reached a consensus, or, if necessary, by consulting a third review author.

**Table 1:** Characteristics of included reviews

Domain	Study, year, and reference	Included study designs	Aim	Dates of searches	Number of studies	Countries	Setting	Population	Intervention	Comparison	Outcomes
Space in SSN units	O'Callaghan *et al.*, 2019 [12]	RCTs, non-RCTs, observational studies, qualitative studies, guideline, systematic review	Identify NICU design features which improve neonatal, parental, and staff outcomes.	2006–16	29	Studies: USA, Brazil, Sweden, Denmark, Turkey, Italy, Taiwan; guidelines: Ireland, UK, USA	NICU	Newborn infants, families, and staff	NICU design features	Comparison group, unspecified	Impact of NICU design features upon newborn infants, their families, and staff
Space in SSN units	Zana-Taieb *et al.*, 2022 [13]	Observational studies, expert opinion, qualitative studies	Provide recommendations regarding the minimum area required for a hospital room for a single neonate and their family.	2011–21	11 (articles: n = 4; guidelines: n = 7)	France, Spain, Sweden, UK, USA, Europe	NICU	Newborn infants and their parents and caregivers	Minimum NICU room size	NR	Optimal surface area for a room; organisation of a room, strategies for implementing these recommendations
Health workforce ratios	Bosak *et al.*, 2023 [14]	Simulation studies, any assessment of HWFP models	Identify the approaches (dimensions) and components of HWFP models using a systematic review.	2004–21	20	Canada, England, Iran, Italy, Kazakhstan, Saudi Arabia, South Korea, Thailand, USA, Zambia	NR	NR	Forecasting models for health workforce planning	NR	Approaches, components, summary
Health workforce ratios	Lee *et al.*, 2024 [15]	Quantitative study designs	Systematically review and synthesise the existing literature on methods used in health workforce projection models to identify key findings, trends, and methodological approaches; critically assess the methodological quality, strengths, and weaknesses of the existing literature, with a focus on its relevance in answering policy and scenario analysis; develop good practice reporting guidelines that encompass essential elements of study design, data, methodology, and reporting specific to health workforce projection models.	2010–23	40	Australia, Canada, Germany, Ghana, Guinea, Ireland, Jamaica, Japan, Kazakhstan, Korea, Lesotho, Malawi, New Zealand, Portugal, Saudi Arabia, Serbia, Singapore, Spain, Thailand, UK, USA, OECD countries	Health sector	Focus on workforce planning and projection in the health sector; any population, no limitation on country	Quantitative model for estimating current and future health workforce requirements, estimate demand for and supply, and requirements of workforce	Studies involving quantitative analysis and models that forecast, or project future health workforce needs	Methodological approaches for health workforce projection and forecasting, their strengths and weaknesses, and their relevance in answering policy and scenario analysis
Health workforce ratios	Lopes *et al.*, 2021 [16]	Full text articles	Provide a systematic review of the literature surrounding the modelling of HRH demand and intends to answer the following research questions: How has HRH demand been previously addressed in real-world applications? What are the advantages and barriers of modelling demand for HRH?	Inception to 2019	53	Argentina, Australia, Canada, England, Germany, Israel, Italy, Jamaica, Japan, Netherlands, Portugal, Singapore, South Korea, Sri Lanka, Sweden, Taiwan, Thailand, UK, USA	NR	Health workforce	Modelling of HRH demand	NR	Real-world applications addressing HRH demand, advantages and barriers of modelling demand for HRH
Health workforce ratios	Rafiei *et al.*, 2019 [17]	Reports, books, review articles, qualitative and quantitative studies	Provide a comprehensive overview toward human resource forecasting approaches and consequently propose the features that potentially improve the effectiveness of such an approach.	1970–2014	128	NR	Health system unspecified	All health care professionals such as: doctors, dentists, nurses, dieticians and pharmacists	Conceptual and analytical approaches in health manpower forecasting, methods to estimate health manpower supply and demand	NR	Conceptual approaches, models, and theories
Health workforce ratios	Sharma *et al.*, 2020 [18]	All published data	Provide a critical analysis of existing national nurse staffing norms by comparing with international norms, guidelines and legislations and discussion with national and international research evidence.	1947–2020	20 (statutory bodies: n = 4; research evidence n = 7; international norms n = 9)	Research evidence: India; international norms: Australia, Canada, UK, USA	NSICU, NICU	Nurses	Required nurse-to-patient ratios	Existing nurse-to-patient ratios	Ideal nurse to patient ratio and nurse staffing norms
Time to Travel	Malouf *et al.*, 2020 [19]	RCT, non-RCT, observational studies, mixed-methods studies	Systematically identify, critically appraise and synthesise the evidence relating to: the effect of OU closures on maternal and neonatal outcomes (compared with the surrounding area or a comparable population) and the association between travel distance or time to an OU and maternal and neonatal outcomes.	1990–2019	31 (travel distance and/or time: n = 21)	Canada, Finland, France, Japan, Netherlands, Norway, UK	OU, NICU	Women giving birth in high-income, OECD countries with UHC of maternity services comparable to the UK	Travel distance or travel time to an OU	Comparison group, unspecified	Maternal outcomes: maternal mortality, caesarean section (overall, emergency or intrapartum), severe perineal trauma (including third haemorrhage, maternal admission to ICU, maternal blood transfusion. Neonatal outcomes: stillbirth (overall or intrapartum), neonatal mortality, perinatal mortality, infant mortality, babies born before arrival, neonatal unit admission, Apgar score, hypoxic-ischaemic encephalopathy.

HRH – human resources for health, NR – not reported, NSICU – neonatal surgical intensive care unit, OECD – Organisation for Economic Co-operation and Development, OU – obstetric units, RCT – randomised controlled trial, UHC – universal health coverage

**Table 2:** Results and recommendations from systematic reviews of space requirements in SSN units

			Results		
**Study, year, and reference**	**Included data**	**SSN unit level**	**Single family room**	**Open bay SSN unit**	**Recommendations**
O'Callaghan *et al.*, 2019 [12]	Guidelines	NICU unspecified	Infant space: minimum 15.3 m^2^/cot (164.7 ft^2^) clear floor area; 20–24m^2^/cot (215.3–258.3 ft^2^) recommended in practice.	Individual infant space: minimum 11.2–12 m^2^/cot (120.6–129.2 ft^2^) of clear floor area; 20 m^2^/cot (215.3 ft^2^) when inclusive of access and support space. Multi-cot area infant space: 13.5 m^2^/cot (145.3 ft^2^) core clinical space, includes 0.6 m (2 ft) access space with a minimum distance of 2.4 m (7.9 ft) between cots.	Overall: 20–24 m^2^/cot (215.3–258.3 ft^2^). Single cot areas: 20 m^2^/cot (215.3 ft^2^) inclusive of core cot, access and support space. Multi-cot areas: 13.5 m^2^/cot (145.3ft^2^) core clinical space (4.13 m ×3.27 m +0.6 m (13.5 ft ×10.7ft +2 ft) access space).
Zana-Taieb *et al.*, 2022[13]	Simulation study, user experience, expert recommendation	NICU unspecified	Infant space: minimum 15.3–24 m^2^ (164.7–258.3 ft^2^) floor area (n = 7 studies); minimum 28m^2^ (301.4 ft^2^) if combined mother–baby care (n = 1 studies).	Multi-cot room infant space: minimum 11.2-18.5 m^2^/cot (120.6–199.1 ft^2^) clear floor area (n = 7 studies), additional 10m^2^ (107.6 ft^2^) family area (n = 1 study): Intensive care: 20–24 m^2^/cot (215.3–258.3 ft^2^) (n = 1 study). Special care: 9.5 m2/cot +3 m distance between cots (102.2 ft^2^ + 9.8 ft) (n = 1 study); Routine care: 56 m^2^/4 cots (602.8 ft^2^/4 cots) (n = 1 study). Open-bay unit infant space: minimum 14 m^2^ (+1.2 m clear aisle) – 18.5m^2^/cot (150.7 ft^2^ (+4 ft) – 199.1 ft^2^) (n = 6 studies), minimum 2.4 m (7.9 ft) distance between cots (n = 4 studies), additional 10 m^2^ (107.6 ft^2^) family area (n = 1 study).	Single-family room infant space: minimum of 24m^2^/cot (258.3 ft^2^). Open-bay unit infant space: minimum of 18.5 m^2^/cot (199.1 ft^2^) + 2.4m (7.9 ft) for passage of medical devices and/or mother's bed.

NICU – newborn intensive care unit, SSN – small and sick newborn

### Assessment of methodological quality of included reviews

Two review authors (GW, DS) assessed the methodological quality of the systematic reviews using the Risk of Bias in Systematic Reviews (ROBIS) [20] tool and used a third author to reach consensus if needed. The ROBIS tool has three categories or ‘phases’. The first assesses the relevance of the systematic review, while the second focusses on the review process and has four domains (study eligibility, identification and selection of studies, data collection and study appraisal, and synthesis of findings). The final phase assesses the overall risk of bias based on the interpretation of review findings and limitations. The ROBIS has three final risk of bias ratings:

– low risk of bias (the findings of the review are likely to be reliable, there are no concerns with the review process, or concerns were appropriately considered in the review conclusions, *i.e.* were supported by the evidence and included consideration of the relevance of included studies);

– high risk of bias (one or more of the concerns raised during the assessment was not addressed in the review conclusions, the review conclusions were not supported by the evidence, or the conclusions did not consider the relevance of the included studies to the review question);

– unclear risk of bias (there is insufficient information reported to make a judgement on risk of bias).

We also assessed if the reviews used a standard systematic approach, tool, or checklist for assessing the certainty of the body of evidence synthesised (such as the Grading of Recommendations Assessment, Development, and Evaluation (GRADE) framework [21]) and for developing recommendations (such as the Developing and Evaluating Communication strategies to support Informed Decisions and practice based on Evidence (DECIDE) approach [22]).

### Assessment of overlapping systematic reviews

For each norm with ≥2 systematic reviews, we assessed total and pairwise overlap of primary studies included in said reviews using the correct covered area method and the Graphical Representation of Overlap for OVErviews (GROOVE) tool [23]. We also accounted for ‘chronological structural missingness’ (*i.e.* where a primary study is published after the date of searches in the review, *e.g.* a review published in 2015 cannot include a primary study published in 2018) using GROOVE methods [23]. We used the overlap thresholds described by Pieper and colleagues [24] for interpretation, whereby overlaps of <5%, 5% to <10%, 10% to <15%, and ≥15% indicated slight, moderate, high, and very high overlap, respectively. We did not account for other types of structural missingness (*e.g.* language structural missingness).

### Data analysis

We described the data narratively, summarising each systematic review by question type, date of data collection and outcomes. Where possible, we also described the data by type of study (RCTs, non-randomised studies of interventions, other observational), estimates (*i.e.* incidence, prevalence, means, medians, other); health facility type (health clinic (level 1), district hospital (level 2), tertiary specialised hospital (level 3)); WHO geographic region (African Regional Office (AFRO), Regional Office of the Americas (AMRO), Eastern Mediterranean Regional Office (EMRO), European Regional Office (EURO), South East Asian Regional Office (SEARO), Western Pacific Regional Office (WPRO)) [25]; income band, per World Bank criteria [26] (high income countries (HIC), upper middle-income countries (UMIC), lower middle-income countries (LMIC), low income countries (LIC)); and risk of bias assessment [20]. We also listed any recommendations made by the authors.

## RESULTS

We retrieved 9110 records in the searches for the four overviews. After removing duplicates and screening titles, abstracts, and full texts, we screened 6696 papers and included eight reviews (**Table 1**, **Table 2**, **Table 3**, **Table 4**; Tables S5 and S6 in the **Online Supplementary Document**): two were related to space norms [12,13], five to health workforce norms [14-18], and one to travel time norms [19]). There were two systematic reviews of RCTs [12,13], while the remainder included observational studies or pooled estimates of means, medians, or prevalence data.

**Table 3:** Results and recommendations from systematic reviews of health workforce requirements in SSN units

Study, year, and reference	Included data	Included health workforce models	Acuity level/ward	Nurse-to-patient ratios	Medical ratios/coverage	Summary and recommendations
Bosak *et al.*, 2023 [14]	Simulation studies, any assessment of HWFP models	Conceptual approaches: demand-based, supply-based, mixed supply- and demand-based, target-based, needs-based, and benchmarking. Analytical approaches: system dynamics, simulation, regression, WISN, and artificial neural network.	NA	NA	NA	Summary: identified methods of HWF planning models, which included conceptual and analytical forecasting approaches, each with specific strengths and weaknesses. Recommendations: consider the conditions and context of the country prior to selecting a model or combination of models.
Lee *et al.*, 2024 [15]	Quantitative study designs	Demand analysis: population-to-provider ratio model, utilisation-based model, need-based model, skill-mixed model. Supply analysis: Stock-and-flow model, agent-based simulation model, system dynamic model. Budgetary analysis.	NA	NA	NA	Summary: identified eight different HWF projection models types covering supply, demand, and budgetary components which were frequently used in combination. Recommendations: proposed 30-item ‘Good Practice Reporting Guideline for Health Workforce Models’ in peer-reviewed journals.
Lopes *et al.*, 2021 [16]	Full text articles	Straightforward models. Regression methods: linear regression, autoregressive models, multivariate regression model. Simulation-based methods: System dynamics, Monte Carlo Simulation, Microsimulation, Other simulation models	NA	NA	NA	Summary: presents the advantages and disadvantages of the included methods, the benefits of each method HRH planning, and implementation challenges and issues. Recommendations: future research is needed to: enhance quality and quantity of HRH drivers’ databases; extend research to other health care providers; address HRH demand at a territorial level; and develop modelling methods that are acceptable to both methodologists and policymakers.
Rafiei *et al.*, 2019 [17]	Reports, books, review articles, qualitative and quantitative studies	Conceptual approaches: manpower to population ratio, demand-based approach (requirement model or utilisation-based approach), need based (epidemiological) approach, benchmarking, service target approach, mixed approach (health care utilisation ratio, effective demand-based approach, dynamic system-based framework, effective infrastructure approach, integrating Markov population model). Analytical approaches: extrapolative methods including trend analysis, explanatory variable methods such as econometric models and regression analysis, artificial neural networks, operations research methods including Markov model, linear and nonlinear programming, queuing theory, simulation and system dynamic modelling, WISN.	NA	NA	NA	Summary: provides definitions of each forecasting method, and associated strengths and weaknesses. Recommendations: system dynamic modelling which meets the following features: incorporating all relative factors (using high quality longitudinal data) with dominant effect on manpower supply and demand (such as emerging technologies, socio cultural evolutions, economic changes, political incentives, trends on health system capacity), monitoring trends, considering complex interactions, and applying what if scenarios.
Sharma *et al.*, 2020 [18]	All published data	Activity analysis; ‘Work measurement’ concepts	NICU unspecified	Existing nurse-to-patient ratios in India: non-ventilated and non-surgical patients – 1:10; non-ventilated patients – 1:3.6; ventilated patients – 1:2.4	NR	Summary: there is a need to update the staffing norms in India as they are behind international norms and required ratios estimated by studies conducted in India. National Accreditation Board for Hospitals & Healthcare Providers (NABH) recommendations are the most recent, practical and feasible for use in India. Recommendations: Nurse-to-patient ratio for NICU staffing in India – 1:1. Required nurse-to-patient ratios in India: non-ventilated and non-surgical patients – 1:3.3; non-ventilated patients – 1:3; ventilated patients – 1:2.
			NSICU	NR	NR	Required nurse-to patient ratios in India: intensive care unit – 1.5:1; recovery unit – 1:1; private unit – 1:1.

HRH – human resources for health, HWF – health workforce, ICU – intensive care unit, NA – not applicable, NR – not reported, NSICU – neonatal surgical intensive care unit, WISN – workload indicators of staffing needs

**Table 4:** Results and recommendations from systematic reviews of travel time to health facilities

Study, year, and reference	Outcome	Included data	NICU level	Analysis	Travel distance	Travel time
Malouf *et al.*, 2020 [19]	Neonatal mortality	Observational studies	OU	Narrative analysis	Two studies discussed neonatal mortality and travel distance. No effect size provided. One study discussed neonatal mortality for those born before arrival and travel distance.	Five studies discussed neonatal mortality and travel time. No effect size provided. One study discussed mortality and/or Apgar <4 at five minutes and/or transfer to NICU and travel time.
	Stillbirth (overall or intrapartum)	Observational studies	OU	Narrative analysis	Two studies discussed stillbirth and travel distance. No effect size provided.	Three studies discussed stillbirth and travel time. No effect size provided.
	Perinatal mortality	Observational studies	OU	Narrative analysis	One study discussed perinatal mortality and travel distance. No effect size provided.	Seven studies discussed perinatal mortality and travel time. No effect size provided.
	Infant mortality	Observational studies	OU	Narrative analysis	No studies for this exposure.	One study discussed infant mortality and travel time. No effect size provided.
	Born before arrival	Observational studies	OU	Narrative analysis	Four studies discussed born before arrival and travel distance. No effect size provided.	Six studies discussed born before arrival and travel time. No effect size provided.
	Neonatal unit admission	Observational studies	OU	Narrative analysis	One study discussed neonatal unit admission and travel distance. No effect size provided.	Three studies discussed neonatal unit admission and travel time. No effect size provided.
	Apgar score	Observational studies	OU	Narrative analysis	No studies for this exposure.	One study discussed Apgar score and travel time. No effect size provided.
	Hypoxic-ischemic encephalopathy	Observational studies	OU	Narrative analysis	No studies for this exposure.	No studies for this exposure.
	Maternal mortality	Observational studies	OU	Narrative analysis	One study discussed maternal mortality and travel distance. No effect size provided.	No studies for this exposure.
	Caesarean section (overall or intrapartum)	Observational studies	OU	Narrative analysis	One study discussed caesarean section and travel distance. No effect size provided.	Five studies discussed caesarean section and travel time. No effect size provided.
	Emergency caesarean section	Observational studies	OU	Narrative analysis	One study discussed emergency caesarean section and travel distance. No effect size provided.	One study discussed emergency caesarean section and travel time. No effect size provided.
	Severe perineal trauma	Observational studies	OU	Narrative analysis	No studies for this exposure.	No studies for this exposure.
	Postpartum haemorrhage	Observational studies	OU	Narrative analysis	No studies for this exposure.	Two studies discussed postpartum haemorrhage and travel time. No effect size provided.
	Maternal admission to ICU	Observational studies	OU	Narrative analysis	No studies for this exposure.	One study discussed maternal admission to an ICU and travel time. No effect size provided.
	Maternal blood transfusion	Observational studies	OU	Narrative analysis	No studies for this exposure.	One study discussed maternal blood transfusion and travel time. No effect size provided.

ICU – intensive care unit, OU – obstetric unit

There was a total of 332 primary studies in the eight reviews (median = 30, interquartile range = 20–43; mean = 42, standard deviation = 37). There was a slight overlap in the primary studies included in the reviews on space (4.17%) and health workforce (3.29%) norms, after adjusting for chronological missingness (Figure S6 and Table S7 in the **Online Supplementary Document**). All reviews included data from SSNs aged up to 28 days.

Countries from all WHO regions were included in the reviews. However, AFRO [14,15], SEARO [16,18], and EMRO [14,15] countries were only included in two reviews each, WPRO countries were included in five reviews [12,14-16,18]; while AMRO and EURO countries were included in seven reviews [12–16,18,19]. One review [17] did not specify the included countries.

Only one review [15] included countries of all income levels, *i.e.* HICs, UMICs, LMICs, and LICs. One review [14] included HIC, UMICs, and a LMIC. One review [18] included HICs and an LMIC. Two reviews [12,16] included HICs and UMICs. Only one review [15] included LICs, three included LMICs [14,15,18], five included UMICs [12,14–16,18], and seven included HICs [12–16,18,19]. Two reviews [13,19] included HICs only.

Three reviews [12,13,18] included data from neonatal intensive care units (NICU) only, while one included data from NICUs and maternity wards/obstetric units [19]. The other reviews did not specify the types of health facilities.

Three reviews included studies from inception to the current time [16–18]. One review included studies from the 1990s [19], two included studies from the early 2000s [12,14], while the remainder included studies from 2010 to the current time [15,17].

All reviews included in the space and health workforce domains made recommendations on norms for SSN care (**Table 2**, **Table 3**). The authors of the travel time review were unable to draw firm conclusions due to the heterogeneity of the included studies (**Table 4**).

### Methodological quality

Our ROBIS quality assessment (Figure S7 and Table S7 in the **Online Supplementary Document**) indicated that Malouf 2020 had an low risk of bias overall and across all four domains, study eligibility, identification and selection of studies, data collection and study appraisal, and synthesis of findings. The other seven reviews had high risk of bias both overall and across all domains, except for Sharma 2020, which we deemed to have insufficient information to rate the risk of bias in the domain of study selection.

Only one review [19] assessed risk of bias using a standardised tool – the Newcastle-Ottawa Quality Assessment Scale for Observational Studies [27]. None of the reviews used the Cochrane Risk of Bias Tool 2 or the Cochrane Risk of Bias in Non-Intervention Studies of Interventions. Similarly, none of the reviews reported using a standard tool or checklist for assessing the certainty of the body of evidence (*e.g.* GRADE) or a standard tool for developing recommendations (*e.g.* DECIDE).

## DISCUSSION

Despite the high burden of SSN in LMICs and the need for health facility care for these vulnerable infants, we found only eight systematic reviews that addressed our norms for scaling up SSN care. We also found no other overviews of reviews that examined our research questions.

‘Overviews of reviews’, sometimes called ‘umbrella reviews’ [9,10], are studies designed to map and assess quality of existing systematic reviews in a given area. This means that their aim is not to meta-analyse, pool data, or present quantitative syntheses, but rather to assess if another systematic review is needed in a topic area. We therefore designed our study as an ‘overview of reviews’ to determine if we should commission new systematic reviews to assess the norms for scaling up SSN care.

We found important disparities in reporting in the systematic reviews included in our analysis. Only two of the eight systematic reviews covered AFRO, EMRO and SEARO countries, while seven of them included EURO and AMRO countries. Similarly, only one review included LICs, three included LMICs, and five included UMICs. In contrast, seven reviews encompassed HICs, with two including HICs only. We found no data from level 2 district hospital facilities or level 1 primary health care clinics. Three reviews included data from NICUs only. While one review included data from NICUs and maternity wards/obstetric units. The other reviews did not specify the types of health facilities included.

The eight reviews covered by our analysis were of variable quality. We rated only one review [19] to have an overall low risk of bias, as it used standardised tools and provided details on study eligibility, selection, data collection, and appraisal. The other seven reviews [12–18] were rated as high risk of bias due to lack of standardised reporting and synthesis. There was also little overlap between primary studies in the reviews. Standardised checklists and tools are needed to ensure quality of healthcare evidence and enable results to be compared across studies, countries, and regions [28]. They are also mandated before publication by many journals. However, only Malouf and colleagues [19] used a risk of bias tool (*i.e.* the Newcastle-Ottawa Scale). None of the reviews used a standard tool for assessing the certainty of the body of evidence (*e.g.* GRADE) or a standard tool for developing recommendations (*e.g.* DECIDE).

Our overview had limitations. We reviewed studies published only in the last five years, as we considered that earlier studies would have limited relevance to the development of norms. Furthermore, norms for scaling up of healthcare interventions should include all the health system building blocks (*i.e.* information systems, medicinal products, leadership, governance, and financing [3]) and incorporate measures of both quality of care [6] and access to care [7,8]. However, we limited our research questions only to those posed by the WHO expert group in 2022 (beds, space, health workforce, travel time (**Table 2**)).

For our assessment of the quality of the systematic reviews, we note there were several risk of bias assessments tools recommended by Cochrane Collaboration [20,29,30] including ROBIS [20] and AMSTAR [30], as well as many critical appraisal tools widely used for observational studies, such as the one developed by the Joanna Briggs Institute [31]. We opted for the ROBIS, as it assesses bias in detail and considers complementary domains to AMSTAR 2, which, in turn, assesses bias, but also other aspects of quality which we did not consider in this overview, such as funding source and assessments of conflicts of interest.

The systematic reviews that we located were very heterogeneous. We thus described the data narratively and did not attempt quantitative synthesis, as described by the Cochrane Collaboration and other investigators [9,10].

For space and health workforce, we considered that studies of point or period prevalence, incidence rates, ratios or continuous measures such as means and medians of any estimates would be the most practical method of understanding norms. The included reviews provided these data, though were of high risk of bias and lacked generalisability due to the limited countries included. More detail can be found in the accompanying paper in this series including Time in Motion activites [32], Workforce Indicators of Staffing Need (WISN) [33], and architect design of SSN units to optimise space considerations [12].

For estimates of admission beds, approaches used by the WHO Service Availability and Readiness [34] and United States Agency for International Development Service Provision Assessment [35,36] tools include incidence rate of severe morbidity (admission rates) in combination with length of stay and the catchment population of live births to give the bed days per year. More detail can be found in the paper by Sinha and colleagues [37].

For travel time, the EmOC ‘natural history’ approach [38] (understanding the progression of a disease in an individual over time without any medical intervention, *i.e.* understanding how acute and fulminant a condition is) can assist in understanding ‘how close’ healthcare should be to the patient. The EmOC approach considered the natural history of postpartum haemorrhage in making their recommendation that a health facility should be no more than two hours from a woman’s home [4,5]. For high-risk infants, many neonatologists consider ‘the closer the health care is, the better’, which is why high risk mothers are often transported with their babies *in utero* to tertiary centres. For outborn infants with possible severe morbidity such as fulminating sepsis, many neonatologists consider a 30-minute delay in accessing care as the maximum acceptable limit. Studies of the association between travel time and cause specific mortality, as proposed by the WHO expert panel (**Box 2**), can also be helpful [19]. However, it is well known that these associations could be confounded, *i.e.* that there could be other reasons why an infant who lives further away from a health facility may have a higher risk of neonatal mortality (*e.g.* lower income or education level of a family in a rural area). While effect estimates can be adjusted in these cases, residual confounding remains. Reverse causality is another potential problem, *i.e.* a high-risk mother may move closer to a health facility to reduce her travel time.

Strengths of this review included our rigorous search strategy across multiple databases, the detailed extraction by our research team, and our ROBIS-based quality assessment, all of which minimised bias in our analysis. It is also the first review to report on the landscape of existing reviews and evidence syntheses for four norms needed for SSN care scale up: hospital beds, space, workforce and travel time.

## CONCLUSIONS

In 2010, a global research priority setting exercise identified many research gaps for the care of SSNs [39,40]. Important questions about the efficacy and effectiveness of interventions and the content of care for the SSN have since been addressed [41-43]. However, our overview has shown important disparities and gaps in the global evidence base remain in the ‘how’ to scale up for SSN care.

Due to the lack of high-quality systematic reviews, the WHO and UNICEF have now commissioned four additional systematic reviews. After the systematic reviews are completed, the organisations will proceed to the second project phase to ‘pressure test’ the SSN norms using real life country level data. The results from this work will also be used in future ENAP documents, EmOC re-visioning, and WHO-UNICEF SSN implementation guidance for LMICs [44].

## Additional material


Online Supplementary Document
