# Supplementary material for: Norms for scaling up small and sick newborn care: an overview of reviews
Source: J Glob Health. 2025 Nov 21;15:04290. doi: 10.7189/jogh.15.04290 (PMC12635899; doi:10.7189/jogh.15.04290)
Supplement: Online Supplementary Document [file jogh-15-04290-s001.pdf]

Supplement to: Strobel NA, Whisson G, Swe D, Budrikis A, Edmond KM. Norms for scaling up small and sick newborn care: an overview of reviews. J Glob Health. 2025;15:04290.

Table of Contents

Appendix 1 - Figure S1. PRIOR checklist 2

Appendix 2 - Table S1. Search strategies – Hospital admissions 4

Appendix 3 - Figure S2. PRISMA Flow Diagram – Hospital admissions 9

Appendix 4 - Table S2. Search strategies – Space in NICUs 10

Appendix 5 - Figure S3. PRISMA Flow Diagram – Space in NICUs 12

Appendix 6 - Table S3. Search strategies – Health workforce ratios 13

Appendix 7 - Figure S4. PRISMA Flow Diagram – Health workforce ratios 15

Appendix 8 - Table S4. Search strategies – Time to travel 16

Appendix 9 - Figure S5. PRISMA Flow Diagram – Time to travel 20

Appendix 10 - Table S5. Citations of ongoing studies 21

Appendix 11- Table S6. Excluded studies with reasons 22

Appendix 12 - Figure S6. Graphical Representation of Overlap for OVErviews (GROOVE) considering chronological structural missingness 29

Appendix 13 - Figure S7. ROBIS assessment of the included studies 30

Appendix 1 - Figure S1. PRIOR checklist

PRIOR Checklist

(Gates M, Gates A, Pieper D, et al. Reporting guideline for overviews of reviews of healthcare interventions: development of the PRIOR statement. BMJ 2022;378:e070849. doi:10.1136/bmj-2022-070849)

**SECTION** Topic	**#**	**Item**	**Location reported**
**TITLE**			
Title	1	Identify the report as an overview of reviews	p1
**ABSTRACT**			
Abstract	2	Provide a comprehensive and accurate summary of the purpose, methods, and results of the overview of reviews.	p2
**INTRODUCTION**			
Rationale	3	Describe the rationale for conducting the overview of reviews in the context of existing knowledge.	p3
Objectives	4	Provide an explicit statement of the objective(s) or question(s) addressed by the overview of reviews.	p3
**METHODS**			
Eligibility criteria	5a	Specify the inclusion and exclusion criteria for the overview of reviews. If supplemental primary studies were included, this should be stated, with a rationale.	p4
	5b	Specify the definition of 'systematic review' as used in the inclusion criteria for the overview of reviews.	p4
Information sources	6	Specify all databases, registers, websites, organizations, reference lists, and other sources searched or consulted to identify systematic reviews and supplemental primary studies (if included). Specify the date when each source was last searched or consulted.	p4
Search strategy	7	Present the full search strategies for all databases, registers and websites, such that they could be reproduced. Describe any search filters and limits applied.	p4, Appendix A1,3,5,7
Selection process	8a	Describe the methods used to decide whether a systematic review or supplemental primary study (if included) met the inclusion criteria of the overview of reviews.	p4
	8b	Describe how overlap in the populations, interventions, comparators, and/or outcomes of systematic reviews was identified and managed during study selection.	p4, Appendix 12
Data collection process	9a	Describe the methods used to collect data from reports.	p4,5
	9b	If applicable, describe the methods used to identify and manage primary study overlap at the level of the comparison and outcome during data collection. For each outcome, specify the method used to illustrate and/or quantify the degree of primary study overlap across systematic reviews.	NA
	9c	If applicable, specify the methods used to manage discrepant data across systematic reviews during data collection.	NA
Data items	10	List and define all variables and outcomes for which data were sought. Describe any assumptions made and/or measures taken to identify and clarify missing or unclear information.	p4,5
Risk of bias assessment	11a	Describe the methods used to *assess* risk of bias or methodological quality of the included systematic reviews.	p4,5
	11b	Describe the methods used to *collect* data on (from the systematic reviews) and/or *assess* the risk of bias of the primary studies included in the systematic reviews. Provide a justification for instances where flawed, incomplete, or missing assessments are identified but not re-assessed.	p4,5
	11c	Describe the methods used to *assess* the risk of bias of supplemental primary studies (if included).	NA
Synthesis methods	12a	Describe the methods used to summarize or synthesize results and provide a rationale for the choice(s).	p4,5
	12b	Describe any methods used to explore possible causes of heterogeneity among results.	NA
	12c	Describe any sensitivity analyses conducted to assess the robustness of the synthesized results.	NA
Reporting bias assessment	13	Describe the methods used to *collect* data on (from the systematic reviews) and/or *assess* the risk of bias due to missing results in a summary or synthesis (arising from reporting biases at the levels of the systematic reviews, primary studies, and supplemental primary studies, if included).	p4,5
Certainty assessment	14	Describe the methods used to *collect* data on (from the systematic reviews) and/or *assess* certainty (or confidence) in the body of evidence for an outcome.	p4,5

**RESULTS**			
Systematic review and supplemental primary study selection	15a	Describe the results of the search and selection process, including the number of records screened, assessed for eligibility, and included in the overview of reviews, ideally with a flow diagram.	p5
	15b	Provide a list of studies that might appear to meet the inclusion criteria, but were excluded, with the main reason for exclusion.	App
Characteristics of systematic reviews and supplemental primary studies	16	Cite each included systematic review and supplemental primary study (if included) and present its characteristics.	Table 3,4,5,6
Primary study overlap	17	Describe the extent of primary study overlap across the included systematic reviews.	p5
Risk of bias in systematic reviews, primary studies, and supplemental primary studies	18a	Present assessments of risk of bias or methodological quality for each included systematic review.	p6, Appendix A11
	18b	Present assessments (*collected* from systematic reviews or *assessed* anew) of the risk of bias of the primary studies included in the systematic reviews.	p6, Appendix A11
	18c	Present assessments of the risk of bias of supplemental primary studies (if included).	NA
Summary or synthesis of results	19a	For all outcomes, summarize the evidence from the systematic reviews and supplemental primary studies (if included). If meta-analyses were done, present for each the summary estimate and its precision and measures of statistical heterogeneity. If comparing groups, describe the direction of the effect.	p5,6
	19b	If meta-analyses were done, present results of all investigations of possible causes of heterogeneity.	NA
	19c	If meta-analyses were done, present results of all sensitivity analyses conducted to assess the robustness of synthesized results.	NA
Reporting biases	20	Present assessments (*collected* from systematic reviews and/or *assessed* anew) of the risk of bias due to missing primary studies, analyses, or results in a summary or synthesis (arising from reporting biases at the levels of the systematic reviews, primary studies, and supplemental primary studies, if included) for each summary or synthesis assessed.	p5,6
Certainty of evidence	21	Present assessments (*collected* or *assessed* anew) of certainty (or confidence) in the body of evidence for each outcome.	NA
**DISCUSSION**			
Discussion	22a	Summarize the main findings, including any discrepancies in findings across the included systematic reviews and supplemental primary studies (if included).	p6,7
	22b	Provide a general interpretation of the results in the context of other evidence.	p7,8
	22c	Discuss any limitations of the evidence from systematic reviews, their primary studies, and supplemental primary studies (if included) included in the overview of reviews. Discuss any limitations of the overview of reviews methods used.	p8,9
	22d	Discuss implications for practice, policy, and future research (both systematic reviews and primary research). Consider the relevance of the findings to the end users of the overview of reviews, e.g., healthcare providers, policymakers, patients, among others.	p8,9
**OTHER INFORMATION**			
Registration and protocol	23a	Provide registration information for the overview of reviews, including register name and registration number, or state that the overview of reviews was not registered.	p3
	23b	Indicate where the overview of reviews protocol can be accessed, or state that a protocol was not prepared.	p3
	23c	Describe and explain any amendments to information provided at registration or in the protocol. Indicate the stage of the overview of reviews at which amendments were made.	NA
Support	24	Describe sources of financial or non-financial support for the overview of reviews, and the role of the funders or sponsors in the overview of reviews.	p8
Competing interests	25	Declare any competing interests of the overview of reviews' authors.	p8
Author information	26a	Provide contact information for the corresponding author.	p1
	26b	Describe the contributions of individual authors and identify the guarantor of the overview of reviews.	p8
Availability of data and other materials	27	Report which of the following are available, where they can be found, and under which conditions they may be accessed: template data collection forms; data collected from included systematic reviews and supplemental primary studies; analytic code; any other materials used in the overview of reviews.	p8

Appendix 2 - Table S1. Search strategies – Hospital admissions

Embase Classic+Embase <1947 to 2023 March 14>

#	**Search**
1	epidemiology/
2	exp case control study/
3	cohort analysis/
4	cross-sectional study/
5	case study/
6	follow up/
7	longitudinal study/
8	retrospective study/
9	prospective study/
10	observational study/
11	correlational study/
12	epidemiologic.ab,ti.
13	case control.ab,ti.
14	case referent.ab,ti.
15	case stud$.ab,ti.
16	case series.ab,ti.
17	cohort?.ab,ti.
18	cross sectional.ab,ti.
19	follow up.ab,ti.
20	longitudinal.ab,ti.
21	retrospective$.ab,ti.
22	prospective$.ab,ti.
23	observational.ab,ti.
24	Correlational.ab,ti.
25	ecological stud$.ab,ti.
26	Descriptive stud$.ab,ti.
27	adverse effect?.ab,ti.
28	1 or 2 or 3 or 4 or 5 or 6 or 7 or 8 or 9 or 10 or 11 or 12 or 13 or 14 or 15 or 16 or 17 or 18 or 19 or 20 or 21 or 22 or 23 or 24 or 25 or 26 or 27
29	(infan$ or (newborn or new born or newly born) or (neonat$ or neo nat$) or (baby or babies)).ti,ab.
30	newborn/
31	Infant/
32	29 or 30 or 31
33	exp hospital admission/
34	((admission$ adj3 rate$) or (admission$ adj3 hospital)).mp.
35	(hospitali?ation adj3 rate$).mp.
36	((length adj2 stay) or LOS or ((extended or long or short or brief) adj3 (admission* or hospitali$ation*))).mp.
37	(all adj3 cause$).mp.
38	(duration adj2 (admit$ or admission$)).mp.
39	((episode$ adj3 care) or (hospital$ adj3 episode$)).mp.
40	(incidence$ adj2 rate$).mp. [mp=title, abstract, heading word, drug trade name, original title, device manufacturer, drug manufacturer, device trade name, keyword heading word, floating subheading word, candidate term word]
41	33 or 34 or 35 or 36 or 37 or 38 or 39 or 40
42	systematic review.mp. or "Systematic Review"/
43	meta-analysis.mp. or Meta-Analysis/
44	42 or 43
45	28 and 32 and 41 and 44
46	COVID-19.mp. or coronavirus disease 2019/

47	(rat or rats or mouse or mice or rodent or rodents or swine or porcine or murine or sheep or lamb or lambs or ewe or ewes or pig or pigs or piglet or piglets or sow or sows or rabbit or rabbits or cat or cats or kitten or kittens or dog or dogs or puppy or puppies or monkey or monkeys or horse or horses or foal or foals or equine or calf or calves or cattle or heifer or heifers or hamster or hamsters or chicken or chickens or livestock or panda or pandas or buffalo$ or baboon$).mp.
48	(maternal adj2 (morbidity or mortality or complication or outcomes)).mp.
49	45 not (46 or 47 or 48)
50	limit 49 to yr="2018 -Current"

Ovid MEDLINE(R) ALL <1946 to March 14, 2023>

#	Search
1	epidemiology/
2	exp case control study/
3	cohort analysis/
4	cross-sectional study/
5	case study/
6	follow up/
7	longitudinal study/
8	retrospective study/
9	prospective study/
10	observational study/
11	correlational study/
12	epidemiologic.ab,ti.
13	case control.ab,ti.
14	case referent.ab,ti.
15	case stud$.ab,ti.
16	case series.ab,ti.
17	cohort?.ab,ti.
18	cross sectional.ab,ti.
19	follow up.ab,ti.
20	longitudinal.ab,ti.
21	retrospective$.ab,ti.
22	prospective$.ab,ti.
23	observational.ab,ti.
24	Correlational.ab,ti.
25	ecological stud$.ab,ti.
26	Descriptive stud$.ab,ti.
27	adverse effect?.ab,ti.
28	1 or 2 or 3 or 4 or 5 or 6 or 7 or 8 or 9 or 10 or 11 or 12 or 13 or 14 or 15 or 16 or 17 or 18 or 19 or 20 or 21 or 22 or 23 or 24 or 25 or 26 or 27
29	(infan$ or (newborn or new born or newly born) or (neonat$ or neo nat$) or (baby or babies)).ti,ab.
30	newborn/
31	Infant/
32	29 or 30 or 31
33	((admission$ adj3 rate$) or (admission$ adj3 hospital)).mp.
34	(hospitali?ation adj3 rate$).mp.
35	((length adj2 stay) or LOS or ((extended or long or short or brief) adj3 (admission* or hospitali$ation*))).mp.
36	(all adj3 cause$).mp.
37	(duration adj2 (admit$ or admission$)).mp.
38	((episode$ adj3 care) or (hospital$ adj3 episode$)).mp.
39	(incidence$ adj2 rate$).mp.
40	33 or 34 or 35 or 36 or 37 or 38 or 39

41	systematic review.mp. or "Systematic Review"/
42	meta-analysis.mp. or Meta-Analysis/
43	41 or 42
44	28 and 32 and 40 and 43
45	COVID-19.mp. or coronavirus disease 2019/
46	(rat or rats or mouse or mice or rodent or rodents or swine or porcine or murine or sheep or lamb or lambs or ewe or ewes or pig or pigs or piglet or piglets or sow or sows or rabbit or rabbits or cat or cats or kitten or kittens or dog or dogs or puppy or puppies or monkey or monkeys or horse or horses or foal or foals or equine or calf or calves or cattle or heifer or heifers or hamster or hamsters or chicken or chickens or livestock or panda or pandas or buffalo$ or baboon$).mp.
47	(maternal adj2 (morbidity or mortality or complication or outcomes)).mp.
48	44 not (45 or 46 or 47)

Cochrane Library Date Run: 16/03/2023

**ID**	**Search**
#1	(infan* or newborn or new born or newly born or neonat* or neo nat* or baby or babies)
#2	MeSH descriptor: [Infant, Newborn] explode all trees
#3	#1 OR #2
#4	((admission* NEAR/3 rate*) OR (admission* NEAR/3 hospital))
#5	MeSH descriptor: [Patient Admission] this term only
#6	MeSH descriptor: [Incidence] this term only
#7	(hospitali?ation adj3 rate*)
#8	MeSH descriptor: [Hospitalization] this term only
#9	((length NEAR/2 stay) or LOS or ((extended or long or short or brief) NEAR/3 (admission* or hospitali$ation*)))
#10	(all adj3 cause$)
#11	(duration adj2 (admit$ or admission$))
#12	((episode$ adj3 care) or (hospital$ adj3 episode$))
#13	MeSH descriptor: [Patient Readmission] this term only
#14	#4 OR #5 OR #6 OR #7 OR #8 OR #9 OR #10 OR #11 OR #12 OR #13
#15	MeSH descriptor: [Epidemiologic Studies] explode all trees
#16	MeSH descriptor: [Observational Studies as Topic] this term only
#17	MeSH descriptor: [Observational Study] explode all trees
#18	MeSH descriptor: [Cohort Studies] this term only
#19	MeSH descriptor: [Validation Studies as Topic] this term only
#20	MeSH descriptor: [Cross-Sectional Studies] this term only
#21	(observational near/3 (study or studies or design or analysis or analyses)) or cohort* or (prospective near/7 (study or studies or design or analysis or analyses)) or ((follow up or followup) near/7 (study or studies or design or analysis or analyses)) or ((longitudinal or longterm or (long next term)) near/7 (study or studies or design or analysis or analyses or data)) or (retrospective near/7 (study or studies or design or analysis or analyses or data or review)) or ((case next control) or (case next comparison) or (case next controlled)) or (case-referent near/3 (study or studies or design or analysis or analyses)) or (population near/3 (study or studies or analysis or analyses)) or (descriptive near/3 (study or studies or design or analysis or analyses)) or ((multidimensional or (multi next dimensional)) near/3 (study or studies or design or analysis or analyses)) or (cross next sectional) or ((natural next experiment) or (natural next experiments)) or (quasi next (experiment or experiments or experimental)) or ((non experiment or nonexperiment or non experimental or nonexperimental) near/3 (study or studies or design or analysis or analyses)) or (ecologic$ near/3 (study or studies or analysis or analyses))
#22	#15 OR #16 OR #16 OR #17 OR #18 OR #19 OR #20 OR #21
#23	#3 AND #14 AND #22
#24	(rat or rats or mouse or mice or rodent or rodents or swine or porcine or murine or sheep or lamb or lambs or ewe or ewes or pig or pigs or piglet or piglets or sow or sows or rabbit or rabbits or cat or cats or kitten or kittens or dog or dogs or puppy or puppies or monkey or monkeys or horse

	or horses or foal or foals or equine or calf or calves or cattle or heifer or heifers or hamster or hamsters or chicken or chickens or livestock or panda or pandas or buffalo* or baboon*)
#25	MeSH descriptor: [COVID-19] explode all trees
#26	MeSH descriptor: [Randomized Controlled Trial] explode all trees
#27	MeSH descriptor: [Randomized Controlled Trials as Topic] explode all trees
#28	(maternal near/2 (morbidity or mortality or complication or outcomes))
#29	#23 NOT #24
#30	#29 NOT #28
#31	#30 NOT #26
#32	#31 NOT #27 with Cochrane Library publication date Between Jan 2018 and Dec 2023
#33	Cochrane Reviews only

PROSPERO Date Run: 23 March 2023

Line	Search for
#1	MeSH DESCRIPTOR Infant EXPLODE ALL TREES
#2	MeSH DESCRIPTOR Infant, Newborn EXPLODE ALL TREES
#3	(infan* or newborn or new born or newly born or neonat* or neo nat* or baby or babies)
#4	#3 OR #2 OR #1
#5	((admission* adj3 rate*) OR (admission* adj3 hospital))
#6	MeSH DESCRIPTOR Patient Admission EXPLODE ALL TREES
#7	MeSH DESCRIPTOR Patient Readmission EXPLODE ALL TREES
#8	MeSH DESCRIPTOR Incidence EXPLODE ALL TREES
#9	MeSH DESCRIPTOR Hospitalization EXPLODE ALL TREES
#10	(hospitali*ation adj3 rate*)
#11	(all adj3 cause*) adj4 (admission* or admi* or hopitali*ation*)
#12	(duration adj2 (admit* or admission*))
#13	((episode* adj3 care) or (hospital* adj3 episode*))
#14	(incidence* adj2 rate*)
#15	#5 OR #6 OR #7 OR #8 OR #9 OR #10 OR #11 OR #12 OR #13 OR #14
#16	MeSH DESCRIPTOR Systematic Review EXPLODE ALL TREES
#17	MeSH DESCRIPTOR Systematic Reviews as Topic EXPLODE ALL TREES
#18	MeSH DESCRIPTOR Meta-Analysis as Topic EXPLODE ALL TREES
#19	(systematic review*) or (meta-analys*) or (meta analys*)
#20	#19 OR #18 OR #17 OR #16
#21	#20 AND #15 AND #4
#22	MeSH DESCRIPTOR COVID-19 EXPLODE ALL TREES
#23	(rat or rats or mouse or mice or rodent or rodents or swine or porcine or murine or sheep or lamb or lambs or ewe or ewes or pig or pigs or piglet or piglets or sow or sows or rabbit or rabbits or cat or cats or kitten or kittens or dog or dogs or puppy or puppies or monkey or monkeys or horse or horses or foal or foals or equine or calf or calves or cattle or heifer or heifers or hamster or hamsters or chicken or chickens or livestock or panda or pandas or buffalo* or baboon*)
#24	#21 NOT #22
#25	#24 NOT #23

Epistemonikos Date Run: 24 March 2023

Line	Query
1	(title:((infan* OR newborn OR newly born OR neonat* OR neo nat* OR baby OR babies) OR abstract:(infan* OR newborn OR newly born OR neonat* OR neo nat* OR baby OR babies)) OR abstract:((infan* OR newborn OR newly born OR neonat* OR neo nat* OR baby OR babies) OR abstract:(infan* OR newborn OR newly born OR neonat* OR neo nat* OR baby OR babies)))

2	AND (title:((admission* adj3 rate*) OR (admission* adj3 hospital) or (hospitali*ation adj3 rate*) or ((length adj2 stay) or LOS or ((extended or long or short or brief) adj3 (admission* or hospitali*ation*))) or (all adj3 cause*) or (duration adj2 (admit* or admission*)) or ((episode* adj3 care) or (hospital* adj3 episode*)) or (incidence* adj2 rate*)) OR abstract:((admission* adj3 rate*) OR (admission* adj3 hospital) or (hospitali*ation adj3 rate*) or ((length adj2 stay) or LOS or ((extended or long or short or brief) adj3 (admission* or hospitali*ation*))) or (all adj3 cause*) or (duration adj2 (admit* or admission*)) or ((episode* adj3 care) or (hospital* adj3 episode*)) or (incidence* adj2 rate*)))
3	NOT (title:((rat or rats or mouse or mice or rodent or rodents or swine or porcine or murine or sheep or lamb or lambs or ewe or ewes or pig or pigs or piglet or piglets or sow or sows or rabbit or rabbits or cat or cats or kitten or kittens or dog or dogs or puppy or puppies or monkey or monkeys or horse or horses or foal or foals or equine or calf or calves or cattle or heifer or heifers or hamster or hamsters or chicken or chickens or livestock or panda or pandas or buffalo* or baboon*)) OR abstract:((rat or rats or mouse or mice or rodent or rodents or swine or porcine or murine or sheep or lamb or lambs or ewe or ewes or pig or pigs or piglet or piglets or sow or sows or rabbit or rabbits or cat or cats or kitten or kittens or dog or dogs or puppy or puppies or monkey or monkeys or horse or horses or foal or foals or equine or calf or calves or cattle or heifer or heifers or hamster or hamsters or chicken or chickens or livestock or panda or pandas or buffalo* or baboon*)))
4	NOT (title:(covid-19 or coronavirus-19 or covid 19) OR abstract:(covid-19 or coronavirus-19 or covid 19))
5	[Filters: protocol=no, classification=systematic-review, min_year=2019, max_year=2022]

Appendix 3 - Figure S2. PRISMA Flow Diagram – Hospital admissions

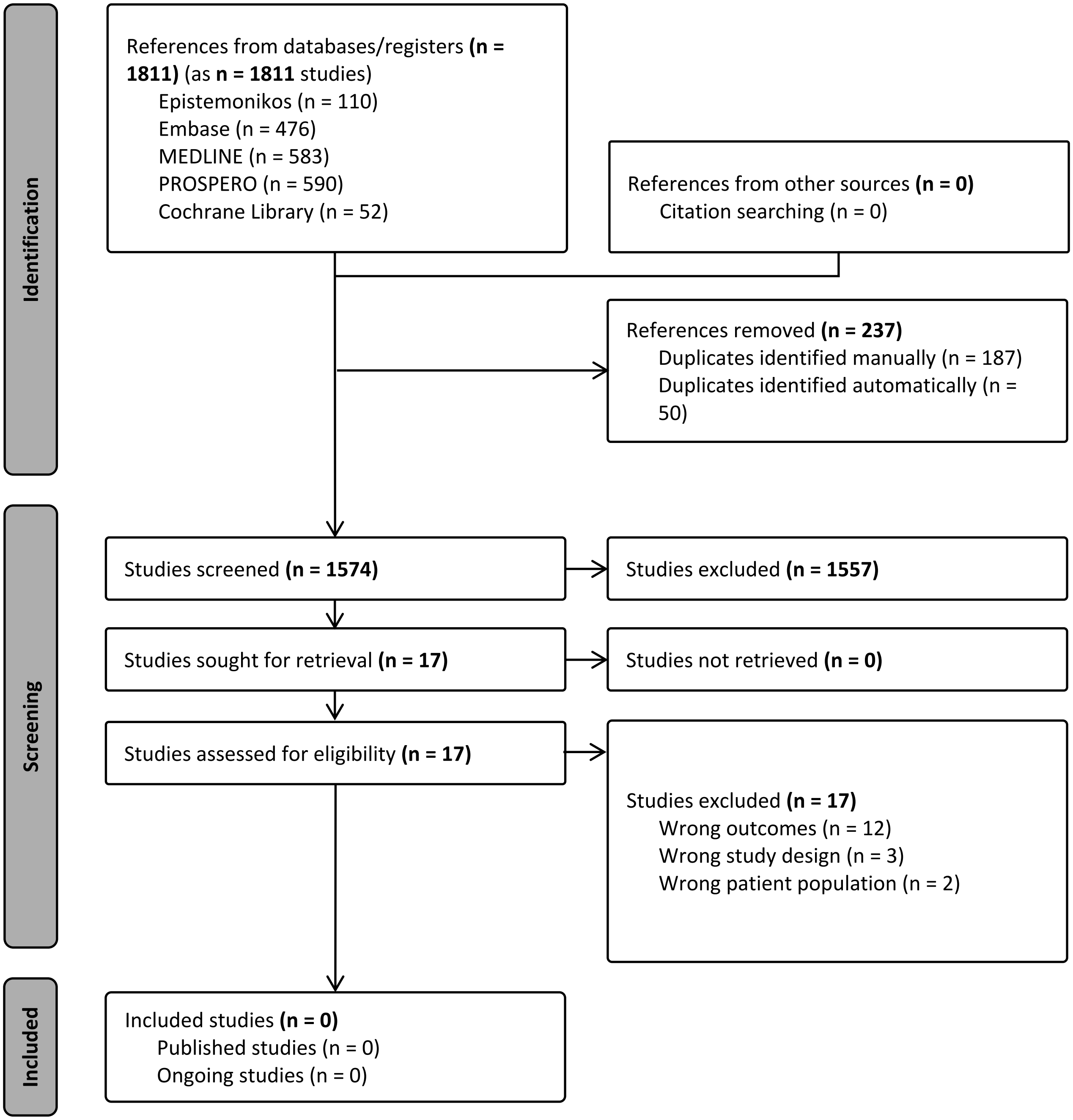

Appendix 4 - Table S2. Search strategies – Space in NICUs

Embase Classic+Embase <1947 to 2023 August 02>

No.	Query
1	(NICU or PICU or SCN).ti,ab,kf.
2	newborn intensive care/ or newborn care/ or neonatal intensive care unit/
3	(((neonat* or neo nat* or p?ediatr*) adj "intensive care unit*") or special care nurser*).ti,ab,kf.
4	1 or 2 or 3
5	(space* or design* or layout* or arrange*).ti,ab,kf.
6	"review"/ or "systematic review"/ or "meta analysis (topic)"/ or meta analysis/ or network meta-analysis/
7	((systematic adj (review$1 or overview$1)) or "literature review" or ("meta analy$3" or "metaanaly$3" or "meta-analy$3")).ti,ab,kw.
8	6 or 7
9	4 and 5 and 8
10	limit 9 to yr="2018 -Current"

Ovid MEDLINE(R) ALL <1946 to August 02, 2023>

No.	Query
1	(NICU or PICU or SCN).ti,ab,kf.
2	(((neonat* or neo nat* or p?ediatr*) adj "intensive care unit*") or special care nurser*).ti,ab,kf.
3	Intensive Care, Neonatal/ or Intensive Care Units, Pediatric/ or Intensive Care Units, Neonatal/
4	1 or 2 or 3
5	(space* or design* or layout* or arrange*).ti,ab.
6	((systematic adj (review$1 or overview$1)) or "literature review" or ("meta analy$3" or "metaanaly$3" or "meta-analy$3")).ti,ab,kw.
7	"Systematic Review"/ or Meta-Analysis/ or Network Meta-Analysis/ or "Review"/
8	6 or 7
9	4 and 5 and 8
10	limit 9 to yr="2018 -Current"

Cochrane Library Date Run: 27/07/2023 03:39:24

No.	Query
#1	(space* or design* or layout* or arrange*):ti,ab,kw
#2	MeSH descriptor: [Intensive Care Units, Neonatal] explode all trees
#3	(((neonat* or neo nat* or pediatr* or paediatr*) NEXT intensive care unit*) or special care nurser* or NICU or PICU or SCN or SSNC):ti,ab,kw
#4	#2 OR #3
#5	#1 AND #4<br>with Cochrane Library publication date Between Jan 2018 and Dec 2023
#6	#5 - Cochrane Reviews

PROSPERO Date run: 27/07/2023

No.	Query
#1	MeSH DESCRIPTOR Intensive Care Units, Neonatal EXPLODE ALL TREES
#2	MeSH DESCRIPTOR Intensive Care, Neonatal EXPLODE ALL TREES
#3	NICU
#4	neonatal intensive care unit
#5	PICU
#6	paediatric intensive care unit
#7	pediatric intensive care unit
#8	SCN

#9	special care nursery
#10	special care nurseries
#11	SSNC
#12	neonatal intensive care
#13	"small and sick newborn care"
#14	"small or sick newborn care"
#15	#1 OR #2 OR #3 OR #4 OR #5 OR #6 OR #7 OR #8 OR #9 OR #10 OR #11 OR #12 OR #13 OR #14
#16	space*
#17	(design*):TI
#18	layout*
#19	arrange*
#20	#19 OR #18 OR #17 OR #16
#21	#20 AND #15

Epistemonikos Date run: 27/07/2023

No.	Query
#1	(title:(((NICU OR PICU OR SCN OR SSNC OR "neonatal intensive care" OR "pediatric intensive care" OR "paediatric intensive care" OR "special care nurser*") AND (design* OR layout* OR space* OR arrange*))) OR abstract:(((NICU OR PICU OR SCN OR SSNC OR "neonatal intensive care" OR "pediatric intensive care" OR "paediatric intensive care" OR "special care nurser*") AND (design* OR layout* OR space* OR arrange*))))
#2	Publication year: Last 5 years
#3	Publication type: Systematic review
#4	#1 AND #2 AND #3

Appendix 5 - Figure S3. PRISMA Flow Diagram – Space in NICUs

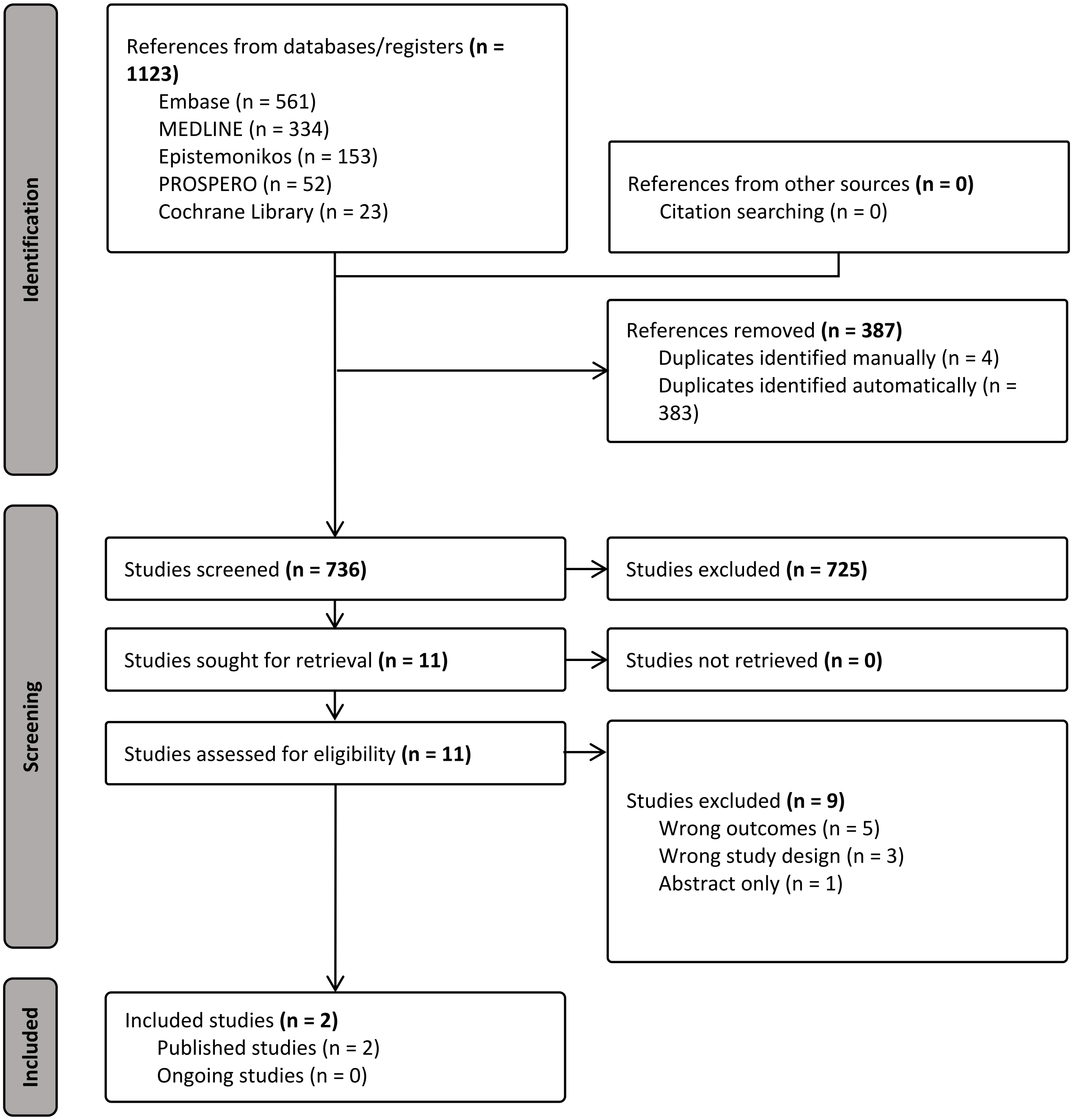

Appendix 6 - Table S3. Search strategies – Health workforce ratios

Embase Classic+Embase <1947 to 2023 November 15>

No.	Query
1	health workforce/
2	(workforce? or staff or staffing or manpower or womanpower or personnel or "human resource?" or "labo?r suppl$3").ti,ab.
3	health*.ti,ab.
4	2 and 3
5	1 or 4
6	workload/ or task performance/ or working time/ or "organization and management"/
7	((Workload indicators adj2 staffing needs) or WISN or "WISN ratio?").ti,ab. or "Workload components".kw.
8	(forecast$3 or estimat$3 or model?ing or model?ed or measure$5).ti,ab.
9	6 or 7 or 8
10	((systematic adj (review$1 or overview$1)) or "literature review" or ("meta analy$3" or "metaanaly$3" or "meta-analy$3")).ti,ab,kw.
11	"review"/ or "systematic review"/ or "meta analysis (topic)"/ or meta analysis/ or network meta-analysis/
12	10 or 11
13	5 and 9 and 12
14	scoping review.ti,ab.
15	13 not 14
16	limit 15 to yr="2018 - Current"

Ovid MEDLINE(R) ALL <1946 to November 15, 2023>

No.	Query
1	Health Workforce/
2	(workforce? or staff or staffing or manpower or womanpower or personnel or "human resource?" or "labo?r suppl$3").ti,ab.
3	health*.ti,ab.
4	2 and 3
5	1 or 4
6	Workload/ or "Task Performance and Analysis"/ or "Personnel Staffing and Scheduling"/
7	((Workload indicators adj2 staffing needs) or WISN or "WISN ratio?").ti,ab. or "Workload components".kw.
8	(forecast$3 or estimat$3 or model?ing or model?ed or measure$5).ti,ab.
9	6 or 7 or 8
10	((systematic adj (review$1 or overview$1)) or "literature review" or ("meta analy$3" or "metaanaly$3" or "meta-analy$3")).ti,ab,kw.
11	"Systematic Review"/ or Meta-Analysis/ or Network Meta-Analysis/ or "Review"/
12	10 or 11
13	5 and 9 and 12
14	scoping review.ti,ab.
15	13 not 14
16	limit 15 to yr="2018 - Current"

Cochrane Library Date Run: 16/11/2023 15:12:44

No.	Query
#1	MeSH descriptor: [Workforce] explode all trees
#2	(workforce? or staff* or manpower or womanpower or personnel or (human NEXT resource?) or (labo#r NEXT suppl*)):ti,ab

#3	health*:ti,ab
#4	#2 AND #3
#5	#1 OR #4
#6	MeSH descriptor: [Health Workforce] explode all trees
#7	MeSH descriptor: [Workload] explode all trees
#8	MeSH descriptor: [Task Performance and Analysis] explode all trees
#9	MeSH descriptor: [Personnel Staffing and Scheduling] explode all trees
#10	("Workload indicators of staffing needs" or WISN):ti,ab,kw
#11	(plan* or forecast* or estimat* or model* or measure*):ti,ab
#12	((nurse? or doctor? or physician? or workforce? or staff*) NEXT/3 (activ* or task* or availabilit*)):ti,ab
#13	#6 OR #7 OR #8 OR #9 OR #10 OR #11 OR #12
#14	#5 AND #13
#15	scoping review:ti,ab
#16	#14 NOT #15
#17	#16 - Cochrane Reviews
#18	#16 - Cochrane Reviews<br>with Publication date Between Jan 2018 and Dec 2023

PROSPERO Date run: 16/11/2023

No.	Query
#1	MeSH DESCRIPTOR Health Workforce EXPLODE ALL TREES
#2	(workforce or workforces or staff or staffing or manpower or womanpower or personnel or "human resource" or "human resources" or "labor suppl*" or "labour suppl*"):TI
#3	health*
#4	#3 AND #2
#5	#4 OR #1
#6	MeSH DESCRIPTOR Workload EXPLODE ALL TREES
#7	MeSH DESCRIPTOR Task Performance and Analysis EXPLODE ALL TREES
#8	MeSH DESCRIPTOR Personnel Staffing and Scheduling EXPLODE ALL TREES
#9	("Workload indicators of staffing needs" or WISN)
#10	(plan or planning or forecast or forcasting or estimate or estimating or estimation or model or models or modeling or modelling or modeled or modelled or measure or measurement):TI
#11	#10 OR #9 OR #8 OR #7 OR #6
#12	#11 AND #5
#13	scoping review
#14	#13 NOT #14

Epistemonikos Date run: 2023/11/16 07:18

No.	Query
#1	(title:((workforce OR workforces OR staff* OR manpower OR womanpower OR personnel OR "human resource" OR "health resources" OR "labor supply" OR "labor supplies" OR "labour supply" OR "labour supplies")) OR abstract:((workforce OR workforces OR staff* OR manpower OR womanpower OR personnel OR "human resource" OR "health resources" OR "labor supply" OR "labor supplies" OR "labour supply" OR "labour supplies")))
#2	(title:((forecast* OR estimat* OR modelled OR modeled OR modelling OR modeling OR WISN OR "workload indicators of staffing needs")) OR abstract:((forecast* OR estimat* OR modelled OR modeled OR modelling OR modeling OR WISN OR "workload indicators of staffing needs")))
#3	(title:(health*) OR abstract:(health*))
#4	#1 AND #2 AND #3
#5	Publication type: Systematic Review

Appendix 7 - Figure S4. PRISMA Flow Diagram – Health workforce ratios

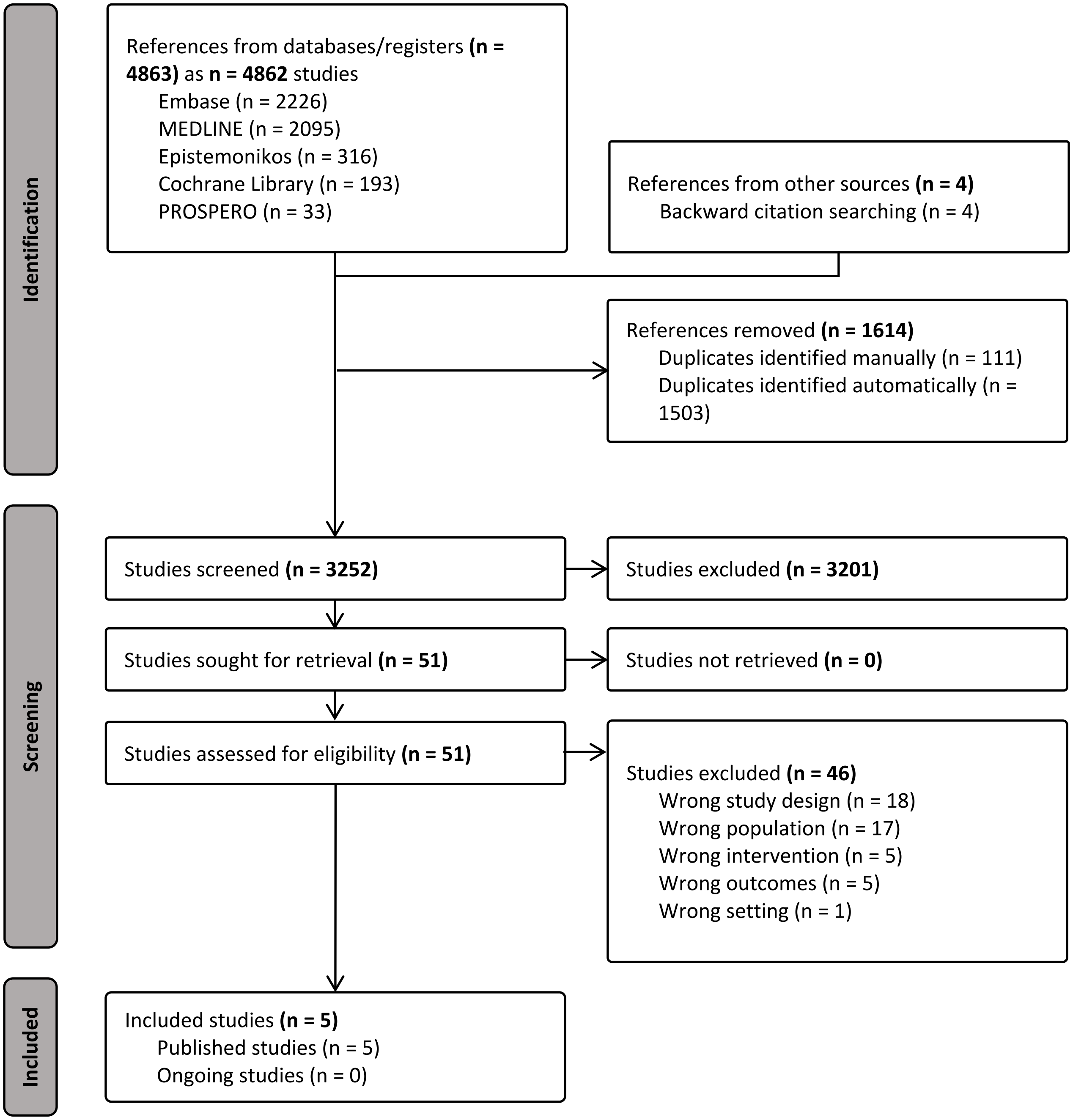

Appendix 8 - Table S4. Search strategies – Time to travel

Embase Classic+Embase <1947 to 2023 July 24>

No.	Query
1	(infan$3 or (newborn or new born or newly born) or (neonat$2 or "neo nat$2") or (baby or babies) or perinatal).ti,ab,kw.
2	perinatal period/ or perinatal care/
3	infant/ or baby/ or newborn/
4	1 or 2 or 3
5	health care access/
6	Travel/
7	health care planning/ or catchment area/
8	(travel* adj3 (distance or time)).ti,ab.
9	(access* adj3 (distance or time)).ti,ab.
10	(geograph* adj3 (distance or time)).ti,ab.
11	("distance to" adj3 (hospital? or service? or unit? or ward? or centre? or center?)).ti,ab.
12	("time to" adj3 (hospital? or service? or unit? or ward? or centre? or center?)).ti,ab.
13	(("travel to" or "travel?ing to") adj3 (hospital? or service? or unit? or ward? or centre? or center?)).ti,ab.
14	(("transport to" or "transported to" or "transportation to") adj3 (hospital? or service? or unit? or ward? or centre? or center?)).ti,ab.
15	(("proximity to" or proxim*) adj3 (hospital? or service? or unit? or ward? or centre? or center?)).ti,ab.
16	(("access to" or accessibility) adj3 (hospital? or service? or unit? or ward? orcentre? or center?)).ti,ab.
17	(geographi* adj5 (hospital? or service? or unit? or ward? or centre? or center?)).ti,ab.
18	(access* or travel* or distance* or transport* or transfer* or proximit* or geographi*).ti.
19	(((level 1 or level i or level one or level 2 or level ii or level two or level 3 or level iii or level three) adj5 (service? or hospital? or ward? or unit? or department? or dept? or centre? or center? or care)) and (babies or baby or newborn* or neonat* or infant* or peri-natal or perinatal or postnatal or post-natal)).ti,ab.
20	5 or 6 or 7 or 8 or 9 or 10 or 11 or 12 or 13 or 14 or 15 or 16 or 17 or 18 or 19
21	(death* or dead or died or mortalit* or surviv*).ti,ab,kf.
22	infant mortality/ or mortality/
23	Survival/
24	21 or 22 or 23
25	"review"/ or "systematic review"/ or "meta analysis (topic)"/ or meta analysis/ or network meta-analysis/
26	((systematic adj (review$1 or overview$1)) or "literature review" or ("meta analy$3" or "metaanaly$3" or "meta-analy$3")).ti,ab,kw.
27	25 or 26
28	4 and 20 and 24 and 27
29	limit 28 to yr="2018 -Current"

Ovid MEDLINE(R) ALL <1946 to July 24, 2023>

No.	Query
1	(infan$3 or (newborn or new born or newly born) or (neonat$2 or "neo nat$2") or (baby or babies) or perinatal).ti,ab,kw.
2	Perinatal Care/ or Infant Care/
3	Infant/ or Infant, Newborn/
4	1 or 2 or 3
5	Health Services Accessibility/

6	Travel/
7	regional health planning/ or "catchment area (health)"/
8	(travel* adj3 (distance or time)).ti,ab.
9	(access* adj3 (distance or time)).ti,ab.
10	(geograph* adj3 (distance or time)).ti,ab.
11	("distance to" adj3 (hospital? or service? or unit? or ward? or centre? or center?)).ti,ab.
12	("time to" adj3 (hospital? or service? or unit? or ward? or centre? or center?)).ti,ab.
13	(("travel to" or "travel?ing to") adj3 (hospital? or service? or unit? or ward? or centre? or center?)).ti,ab.
14	(("transport to" or "transported to" or "transportation to") adj3 (hospital? or service? or unit? or ward? or centre? or center?)).ti,ab.
15	(("proximity to" or proxim*) adj3 (hospital? or service? or unit? or ward? or centre? or center?)).ti,ab.
16	(("access to" or accessibility) adj3 (hospital? or service? or unit? or ward? orcentre? or center?)).ti,ab.
17	(geographi* adj5 (hospital? or service? or unit? or ward? or centre? or center?)).ti,ab.
18	(access* or travel* or distance* or transport* or transfer* or proximit* or time* or geographi*).ti.
19	(((level 1 or level i or level one or level 2 or level ii or level two or level 3 or level iii or level three) adj5 (service? or hospital? or ward? or unit? or department? or dept? or centre? or center? or care)) and (babies or baby or newborn* or neonat* or infant* or peri-natal or perinatal or postnatal or post-natal)).ti,ab.
20	5 or 6 or 7 or 8 or 9 or 10 or 11 or 12 or 13 or 14 or 15 or 16 or 17 or 18 or 19
21	(death* or dead or died or mortalit* or surviv*).ti,ab,kf.
22	Mortality/ or Infant Mortality/
23	Survival/
24	21 or 22 or 23
25	((systematic adj (review$1 or overview$1)) or "literature review" or ("meta analy$3" or "metaanaly$3" or "meta-analy$3")).ti,ab,kw.
26	"Systematic Review"/ or Meta-Analysis/ or Network Meta-Analysis/ or "Review"/
27	25 or 26
28	4 and 20 and 24 and 27
29	limit 28 to yr="2018 -Current"

Cochrane Library Date Run 28/07/2023 05:53:51

No.	Query
#1	MeSH descriptor: [Infant] explode all trees
#2	MeSH descriptor: [Perinatal Care] explode all trees
#3	(infan* or (newborn or new born or newly born) or neonat* or (neo NEXT nat*) or (baby or babies) or perinat* or peri-nat*):ti,ab
#4	#1 or #2 or #3
#5	MeSH descriptor: [Health Services Accessibility] explode all trees
#6	MeSH descriptor: [Travel] explode all trees
#7	MeSH descriptor: [Health Systems Plans] explode all trees
#8	(travel* NEAR/3 (distance or time)):ti,ab
#9	(access* NEAR/3 (distance or time)):ti,ab
#10	(geograph* NEAR/3 (distance or time)):ti,ab
#11	("distance to" NEAR/3 (hospital? or service? or unit? or ward? or centre? or center?)):ti,ab
#12	("time to" NEAR/3 (hospital? or service? or unit? or ward? or centre? or center?)):ti,ab
#13	("travel to" NEAR/3 (hospital? or service? or unit? or ward? or centre? or center?)):ti,ab
#14	("traveling to" NEAR/3 (hospital? or service? or unit? or ward? or centre? or center?)):ti,ab
#15	("travelling to" NEAR/3 (hospital? or service? or unit? or ward? or centre? or center?)):ti,ab
#16	("transport to" NEAR/3 (hospital? or service? or unit? or ward? or centre? or center?)):ti,ab
#17	("transported to" NEAR/3 (hospital? or service? or unit? or ward? or centre? or center?)):ti,ab

#18	("proximity to" NEAR/3 (hospital? or service? or unit? or ward? or centre? or center?)):ti,ab
#19	(proxim* NEAR/3 (hospital? or service? or unit? or ward? or centre? or center?)):ti,ab
#20	("access to" NEAR/3 (hospital? or service? or unit? or ward? or centre? or center?)):ti,ab
#21	(geographi* NEAR/3 (hospital? or service? or unit? or ward? or centre? or center?)):ti,ab
#22	(access* or travel* or distance* or transport* or transfer* or proximit* or time* or geographi*):ti
#23	((("level 1" or "level i" or "level one" or "level 2" or "level ii" or "level two" or "level 3" or "level iii" or "level three") NEAR/5 (service? or hospital? or ward? or unit? or department? or dept? or centre? or center? or care)) and (babies or baby or newborn* or neonat* or infant* or peri-natal or perinatal or postnatal or post-natal)):ti,ab
#24	{OR #5-#23}
#25	(death* or dead or died or mortalit* or surviv*):ti,ab
#26	MeSH descriptor: [Mortality] explode all trees
#27	MeSH descriptor: [Infant Mortality] explode all trees
#28	MeSH descriptor: [Survival] explode all trees
#29	{OR #25-#28}
#30	#4 AND #24 AND #29
#31	Cochrane Reviews
#32	Publication date: 01/01/2018 – 31/12/2023

PROSPERO Date run: 28/07/2023

No.	Query
#1	MeSH DESCRIPTOR Infant EXPLODE ALL TREES
#2	MeSH DESCRIPTOR Perinatal Care EXPLODE ALL TREES
#3	MeSH DESCRIPTOR Infant, Newborn EXPLODE ALL TREES
#4	(infan* or (newborn or new born or newly born) or neonat* or (neo NEXT nat*) or (baby or babies) or perinat* or peri-nat*)
#5	#4 OR #3 OR #2 OR #1
#6	MeSH DESCRIPTOR Health Services Accessibility EXPLODE ALL TREES
#7	MeSH DESCRIPTOR Travel EXPLODE ALL TREES
#8	MeSH DESCRIPTOR Health Systems Plans EXPLODE ALL TREES
#9	(travel* NEAR3 (distance or time))
#10	(access* NEAR3 (distance or time))
#11	(geograph* NEAR3 (distance or time))
#12	("distance to" NEAR3 (hospital* or service* or unit* or ward* or centre* or center*))
#13	("time to" NEAR3 (hospital* or service* or unit* or ward* or centre* or center*))
#14	("travel to" NEAR3 (hospital* or service* or unit* or ward* or centre* or center*))
#15	("traveling to" NEAR3 (hospital* or service* or unit* or ward* or centre* or center*))
#16	("travelling to" NEAR3 (hospital* or service* or unit* or ward* or centre* or center*))
#17	("transport to" NEAR3 (hospital* or service* or unit* or ward* or centre* or center*))
#18	("transported to" NEAR3 (hospital* or service* or unit* or ward* or centre* or center*))
#19	("proximity to" NEAR3 (hospital* or service* or unit* or ward* or centre* or center*))
#20	("access to" NEAR3 (hospital* or service* or unit* or ward* or centre* or center*))
#21	(geographi* NEAR3 (hospital* or service* or unit* or ward* or centre* or center*))
#22	((("level 1" or "level i" or "level one" or "level 2" or "level ii" or "level two" or "level 3" or "level iii" or "level three") NEAR5 (service* or hospital* or ward* or unit* or department* or dept* or centre* or center* or care)) and (babies or baby or newborn* or neonat* or infant* or peri-natal or perinatal or postnatal or post-natal))
#23	#22 OR #21 OR #20 OR #19 OR #18 OR #16 OR #17 OR #15 OR #14 OR #13 OR #12 OR #6 OR #7 OR #8 OR #9 OR #10 OR #11
#24	(death* or dead or died or mortalit* or surviv*)
#25	MeSH DESCRIPTOR Infant Mortality EXPLODE ALL TREES
#26	MeSH DESCRIPTOR Mortality EXPLODE ALL TREES
#27	MeSH DESCRIPTOR Survival EXPLODE ALL TREES

#28	#27 OR #26 OR #25 OR #24
#29	#28 AND #23 AND #5

Epistemonikos Date run: 28/07/2023

No.	Query
#1	(title:(((infan* OR newborn OR new born OR (newly born) OR neonat* OR (neo nat*) OR baby OR babies OR perinat* OR peri-nat*) AND((travel* OR access* OR geograph* OR distance* OR time* OR proxim*) AND (hospital* OR service* OR unit* OR ward* OR centre* OR center*)) AND (death* OR dead OR died OR mortalit* OR surviv*))) OR abstract:(((infan* OR newborn OR new born OR (newly born) OR neonat* OR (neo nat*) OR baby OR babies OR perinat* OR peri-nat*) AND((travel* OR access* OR geograph* OR distance* OR time* OR proxim*) AND (hospital* OR service* OR unit* OR ward* OR centre* OR center*)) AND (death* OR dead OR died OR mortalit* OR surviv*))))
#2	Publication year: Last 5 years
#3	Publication type: Systematic review
#4	#1 AND #2 AND #3

Appendix 9 - Figure S5. PRISMA Flow Diagram – Time to travel

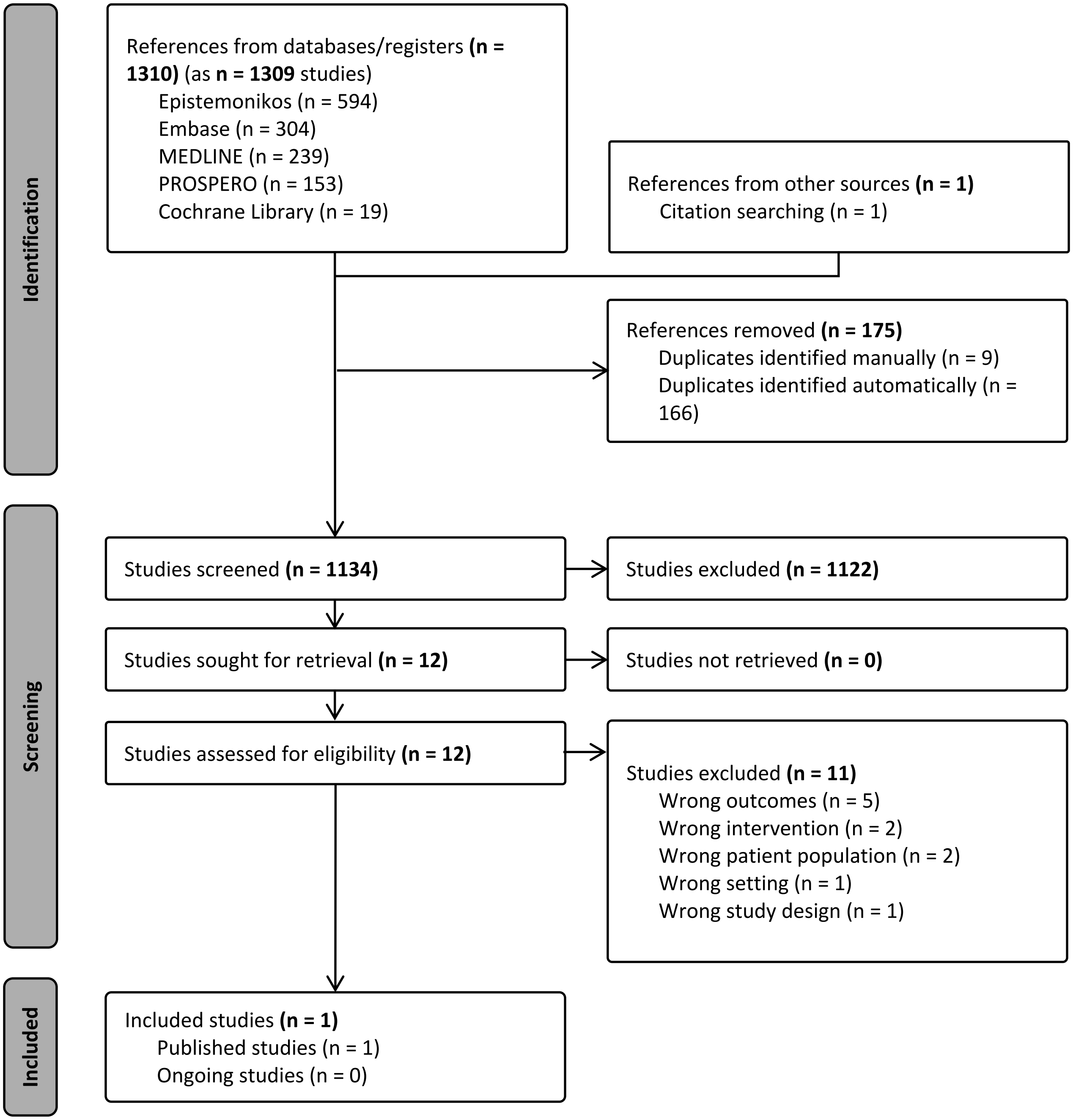

Appendix 10 - Table S5. Citations of ongoing studies

None included.

Appendix 11- Table S6. Excluded studies with reasons

No.	Domain	Citation	Reason for exclusion
1	Hospital admissions	Breidahl, C., Preen, D., & Mozooni, M. (2021). The risk of neonatal intensive care admission and NICU mortality in neonates of international migrants. PROSPERO, CRD42021264003. Retrieved from https://www.crd.york.ac.uk/PROSPERO/view/CRD42021264003	Wrong outcomes
2	Hospital admissions	Isayama, T., Lewis-Mikhael, A.-M., O'Reilly, D., Beyene, J., & McDonald, S. (2021). Health service utilization after postnatal hospital discharge of late preterm versus term infants from infancy through adulthood: a systematic review and meta-analysis. PROSPERO, CRD42016042401. Retrieved from https://www.crd.york.ac.uk/PROSPERO/view/CRD42021264003	Wrong outcomes
3	Hospital admissions	Isayama, T., Lewis-Mikhael, A. M., O'Reilly, D., Beyene, J., & McDonald, S. D. (2017). Health Services Use by Late Preterm and Term Infants From Infancy to Adulthood: A Meta-analysis. Pediatrics, 140(1). doi:10.1542/peds.2017-0266	Wrong outcomes
4	Hospital admissions	Karagiannidou, S., Triantafyllou, C., Zaoutis, T. E., Papaevangelou, V., Maniadakis, N., & Kourlaba, G. (2020). Length of stay, cost, and mortality of healthcare-acquired bloodstream infections in children and neonates: A systematic review and meta-analysis. Infect Control Hosp Epidemiol, 41(3), 342-354. doi:10.1017/ice.2019.353	Wrong outcomes
5	Hospital admissions	Lebreton, E., Crenn-Hebert, C., Menguy, C., Dechartes, A., & Zeitlin, J. (2017). Assessing neonatal morbidity using hospital discharge data: a systematic review. PROSPERO, CRD42017069145. Retrieved from https://www.crd.york.ac.uk/PROSPERO/view/CRD42017069145	Wrong outcomes
6	Hospital admissions	Macy, M. L., Kim, C. S., Sasson, C., Lozon, M. M., & Davis, M. M. (2010). Pediatric observation units in the United States: a systematic review. J Hosp Med, 5(3), 172-182. doi:10.1002/jhm.592	Wrong patient population
7	Hospital admissions	Miller, J. E., Goldacre, R., Moore, H. C., Zeltzer, J., Knight, M., Morris, C., Nowell, S., Wood, R., Carter, K. W., Fathima, P., de Klerk, N., Strunk, T., Li, J., Nassar, N., Pedersen, L. H., Burgner, D. P. (2020). Mode of birth and risk of infection-related hospitalisation in childhood: A population cohort study of 7.17 million births from 4 high-income countries. PLoS Med, 17(11), e1003429. doi:10.1371/journal.pmed.1003429	Wrong study design
8	Hospital admissions	Morche, J., Mathes, T., Jacobs, A., Wessel, L., Neugebauer, E. A. M., & Pieper, D. (2022). Relationship between volume and outcome for gastroschisis: A systematic review. J Pediatr Surg, 57(12), 763-785. doi:10.1016/j.jpedsurg.2022.03.022	Wrong outcomes
9	Hospital admissions	O'Callaghan, N., Dee, A., & Philip, R. K. (2019). Evidence-based design for neonatal units: a systematic review. Matern Health Neonatol Perinatol, 5, 6. doi:10.1186/s40748-019-0101-0	Wrong outcomes
10	Hospital admissions	Phillippi, J. C., Danhausen, K., Alliman, J., & Phillippi, R. D. (2018). Neonatal Outcomes in the Birth Center Setting: A Systematic Review. J Midwifery Womens Health, 63(1), 68-89. doi:10.1111/jmwh.12701	Wrong outcomes
11	Hospital admissions	Royer, A. S., & Busari, J. O. (2021). A systematic review of the impact of intensive care admissions on post discharge cognition in children. Eur J Pediatr, 180(12), 3443-3454. doi:10.1007/s00431-021-04145-5	Wrong outcomes
12	Hospital admissions	Salaeva, D., Tarasoff, L. A., & Brown, H. K. (2020). Health care utilisation in infants and young children born to women with intellectual and developmental disabilities: A systematic review and meta-analysis. J Intellect Disabil Res, 64(4), 303-310. doi:10.1111/jir.12720	Wrong patient population
13	Hospital admissions	Sanderson, M., Sappenfield, W. M., Jespersen, K. M., Liu, Q., & Baker, S. L. (2000). Association between level of delivery hospital and neonatal outcomes among South Carolina Medicaid recipients. Am J Obstet Gynecol, 183(6), 1504-1511. doi:10.1067/mob.2000.107357	Wrong study design
14	Hospital admissions	Seaton, S. (2013). Risk factors for survival and length of stay in neonatal care: a narrative review. PROSPERO, CRD42013006020. Retrieved from https://www.crd.york.ac.uk/PROSPERO/view/CRD42013006020	Wrong outcomes
15	Hospital admissions	Smyth, R. L., Peak, M., Turner, M. A., Nunn, A. J., Williamson, P. R., Young, B., Arnott, J., Bellis, J. R., Bird, K. A., Bracken, L. E., Conroy, E. J., Cresswell, L., Duncan, J. C., Gallagher, R. M., Gargon, E., Hesselgreaves, H., Kirkham, J.	Wrong outcomes

		J., Mannix, H., Smyth, R. M. D., Thiesen, S., Pirmohamed, M. (2014). ADRIC: Adverse Drug Reactions In Children – a programme of research using mixed methods. NIHR Journals Library. Programme Grants for Applied Research, 06, 06. doi:10.3310/pgfar02030	
16	Hospital admissions	Triantafyllou, C., Chorianopoulou, E., Kourkouni, E., Zaoutis, T. E., & Kourlaba, G. (2021). Prevalence, incidence, length of stay and cost of healthcare-acquired pressure ulcers in pediatric populations: A systematic review and meta-analysis. Int J Nurs Stud, 115, 103843. doi:10.1016/j.ijnurstu.2020.103843	Wrong outcomes
17	Hospital admissions	Zona, A., Fazzo, L., Benedetti, M., Bruno, C., Vecchi, S., Pasetto, R., Minichilli, F., De Santis, M., Nannavecchia, A. M., Di Fonzo, D., Contiero, P., Ricci, P., Bisceglia, L., Manno, V., Minelli, G., Santoro, M., Gorini, F., Ancona, C., Scondotto, S., Soggiu, M. E., Scaini, F., Beccaloni, E., Marsili, D., Villa, M. F., Maifredi, G., Magoni, M., Iavarone, I., Gruppo di lavoro SENTIERI 2019-2022. (2023). [SENTIERI - Epidemiological Study of Residents in National Priority Contaminated Sites. Sixth Report]. Epidemiol Prev, 47(1-2 Suppl 1), 1-286. doi:10.19191/EP23.1-2-S1.003	Wrong study design
18	Time to travel	Chambers, D., Baxter, S., & Cantrell, A. (2019). The relationship between distance/travel time to emergency care and patient outcomes. PROSPERO, CRD42019123061. Available from: https://www.crd.york.ac.uk/prospero/display_record.php?ID=CRD42019123061	Wrong patient population
19	Time to travel	Chambers, D., Cantrell, A., Baxter, S., Turner, J., & Booth, A. (2020). Effects of service changes affecting distance/time to access urgent and emergency care facilities on patient outcomes: a systematic review. BMC Med, 18(1), 117. https://doi.org/10.1186/s12916-020-01580-3	Wrong patient population
20	Time to travel	Ehiri, J., Asaolu, I., Alaofe, H., Chebet, J., Esu, E., & Meremikwu, M. (2017). Emergency transportation interventions for reducing adverse pregnancy outcomes in low- and middle-income countries: a systematic review. PROSPERO, CRD42017080092. https://www.crd.york.ac.uk/prospero/display_record.php?ID=CRD42017080092	Wrong intervention
21	Time to travel	Khatri, R. B., & Karkee, R. (2018). Social determinants of health affecting utilisation of routine maternity services in Nepal: a narrative review of the evidence. Reprod Health Matters, 26(54), 32-46. https://doi.org/10.1080/09688080.2018.1535686	Wrong outcomes
22	Time to travel	Lassi, Z. S., Middleton, P., Bhutta, Z. A., & Crowther, C. (2019). Health care seeking for maternal and newborn illnesses in low- and middle-income countries: a systematic review of observational and qualitative studies. F1000Res, 8, 200. https://doi.org/10.12688/f1000research.17828.1	Wrong outcomes
23	Time to travel	Meireles Brito, P., Tavares Netto, A. R., Silva Ribeiro, D. K. S. Q., & Wanick Sarinho, D. S. (2021). Effect of geographical accessibility in the child mortality to the emergency service. PROSPERO, CRD42021279854. https://www.crd.york.ac.uk/prospero/display_record.php?ID=CRD42021279854	Wrong setting
24	Time to travel	Ouma, P. O., Malla, L., Wachira, B. W., Kiarie, H., Mumo, J., Snow, R. W., English, M., & Okiro, E. A. (2022). Geospatial mapping of timely access to inpatient neonatal care and its relationship to neonatal mortality in Kenya. PLOS Glob Public Health, 2(6), e0000216. https://doi.org/10.1371/journal.pgph.0000216	Wrong study design
25	Time to travel	Tekelab, T., Akibu, M., Tagesse, N., Tilhaun, T., Yohanes, Y., & Nepal, S. (2019). Neonatal mortality in Ethiopia: a protocol for systematic review and meta-analysis. Syst Rev, 8(1), 103. https://doi.org/10.1186/s13643-019-1012-x	Wrong outcomes
26	Time to travel	van Lonkhuijzen, L., Stekelenburg, J., & van Roosmalen, J. (2012). Maternity waiting facilities for improving maternal and neonatal outcome in low-resource countries. Cochrane Database Syst Rev, 10(10), CD006759. https://doi.org/10.1002/14651858.CD006759.pub3	Wrong intervention
27	Time to travel	Villamor, E., van Mechelen, K., & Villamor-Martinez, E. (2021). Antenatal and perinatal characteristics and outcome of preterm infants born outside and transported to tertiary specialized perinatal centers (outborn Infants). PROSPERO, CRD42021247005. https://www.crd.york.ac.uk/prospero/display_record.php?ID=CRD42021247005	Wrong outcomes

28	Time to travel	Weldegiorgis, S. K., & Feyisa, M. (2021). Why Women in Ethiopia Give Birth at Home? A Systematic Review of Literature. Int J Womens Health, 13, 1065-1079. https://doi.org/10.2147/IJWH.S326293	Wrong outcomes
29	Space in NICUs	Clapperton, M., Benzies, K., McNeil, D., & Hayden, A. (2021). Neonatal Intensive Care Design and Effect on Infant Health and Development: A Systematic Review. PROSPERO, CRD42021222304. https://www.crd.york.ac.uk/prospero/display_record.php?ID=CRD42021222304	Wrong outcomes
30	Space in NICUs	Denham, M. E., Bushehri, Y., & Lim, L. (2018). Through the Eyes of the User: Evaluating Neonatal Intensive Care Unit Design. HERD, 11(3), 49-65. https://doi.org/10.1177/1937586718761017	Wrong study design
31	Space in NICUs	Kuhn, P., Sizun, J., Casper, C., & Society, G. s. g. f. t. F. N. (2018). Recommendations on the environment for hospitalised newborn infants from the French neonatal society: rationale, methods and first recommendation on neonatal intensive care unit design. Acta Paediatr, 107(11), 1860-1866. https://doi.org/10.1111/apa.14501	Wrong outcomes
32	Space in NICUs	Lall, A., Rabe, H., & Ivashikina, N. (2023). Resource use and costs of neonatal intensive care: a systematic review of economic evidence. CRD42023385198. https://www.crd.york.ac.uk/prospero/display_record.php?ID=CRD42023385198	Wrong outcomes
33	Space in NICUs	Noya, A., Yamaji, N., & Ota, E. (2016). Impact of design on patients in neonatal intensive care units: a systematic review. PROSPERO, CRD42016051086. https://www.crd.york.ac.uk/prospero/display_record.php?ID=CRD42016051086	Wrong outcomes
34	Space in NICUs	O'Callaghan, N., Dee, A., & Philip, R. K. (2019). P480 Evidence-based design for neonatal units: a systematic review. Archives of Disease in Childhood 104(Supplement 3), A344-A345. https://doi.org/10.1136/archdischild-2019-epa.816	Abstract only
35	Space in NICUs	O'Callaghan, N., Dee, A., & Philip, R. K. (2019). P480 Evidence-based design for neonatal units: a systematic review. Archives of Disease in Childhood 104(Supplement 3), A344-A345. https://doi.org/10.1136/archdischild-2019-epa.816	Wrong outcomes
36	Space in NICUs	Villeneuve, E., Landa, P., Allen, M., Spencer, A., Prosser, S., Gibson, A., Kelsey, K., Mujica-Mota, R., Manktelow, B., Modi, N., Thornton, S., & Pitt, M. (2018). A framework to address key issues of neonatal service configuration in England: the NeoNet multimethods study. Health Services and Delivery Research, 6(35). https://doi.org/10.3310/hsdr06350	Wrong study design
37	Space in NICUs	White, R. D., & Consensus Committee on Recommended Design Standards for Advanced Neonatal, C. (2020). Recommended standards for newborn ICU design, 9th edition. J Perinatol, 40(Suppl 1), 2-4. https://doi.org/10.1038/s41372-020-0766-2	Wrong study design
38	Health workforce ratios	Anderson, J., Apaydin, E., Rahman, B., & Sonnen, P. (2021). Staffing Models in Specialty Care. PROSPERO, CRD42021285060. https://www.crd.york.ac.uk/prospero/display_record.php?ID=CRD42021285060	Wrong setting
39	Health workforce ratios	Assaye, A. M., Wiechula, R., Schultz, T., & Feo, R. (2019). Impact of nurse staffing on patient and nurse workforce outcomes in acute care settings in low- and middle-income countries: a systematic review protocol. PROSPERO, CRD42019119428. https://www.crd.york.ac.uk/prospero/display_record.php?ID=CRD42019119428	Wrong population
40	Health workforce ratios	Assaye, A. M., Wiechula, R., Schultz, T. J., & Feo, R. (2021). Impact of nurse staffing on patient and nurse workforce outcomes in acute care settings in low- and middle-income countries: a systematic review. JBI Evid Synth, 19(4), 751-793. https://doi.org/10.11124/JBISRIR-D-19-00426	Wrong intervention
41	Health workforce ratios	Bae, S. H. (2021). Intensive care nurse staffing and nurse outcomes: A systematic review. Nurs Crit Care, 26(6), 457-466. https://doi.org/10.1111/nicc.12588	Wrong population
42	Health workforce ratios	Barrientos Trigo, S., Porcel Gálvez, A. M., Fernández García, E., Abril González, C., & Romero Castillo, R. (2016). Relationship between nurse:patient ratio and staff outcomes in acute care hospital: systematic review. PROSPERO, CRD42016048376. https://www.crd.york.ac.uk/prospero/display_record.php?ID=CRD42016048376	Wrong intervention
43	Health workforce ratios	Bourgon Labelle, J., Audet, L. A., Farand, P., & Rochefort, C. M. (2019). Are hospital nurse staffing practices associated with postoperative cardiac events	Wrong population

		and death? A systematic review. PLOS ONE, 14(10), e0223979. https://doi.org/10.1371/journal.pone.0223979	
44	Health workforce ratios	Butler, M., Schultz, T. J., Halligan, P., Sheridan, A., Kinsman, L., Rotter, T., Beaumier, J., Kelly, R. G., & Drennan, J. (2019). Hospital nurse-staffing models and patient- and staff-related outcomes. Cochrane Database Syst Rev, 4(4), CD007019. https://doi.org/10.1002/14651858.CD007019.pub3	Wrong population
45	Health workforce ratios	Caluwaerts, A., Pintelon, L., & Hopman, J. (2021). Determining staffing levels for environmental cleaning: Time measurements in non-critical care units in an acute care hospital in Brussels. Antimicrobial Resistance and Infection Control, 10(SUPPL 1). https://doi.org/https://dx.doi.org/10.1186/s13756-021-00974-z0	Wrong study design
46	Health workforce ratios	Dall'Ora, C., Rubbo, B., Saville, C., Turner, L., Ball, J., Ball, C., & Griffiths, P. (2023). The association between multi-disciplinary staffing levels and mortality in acute hospitals: a systematic review. Hum Resour Health, 21(1), 30. https://doi.org/10.1186/s12960-023-00817-5	Wrong intervention
47	Health workforce ratios	Dejaco, C., Lackner, A., & Sprenger, M. (2014). Methods and models used to estimate the needs of rheumatology manpower. PROSPERO, CRD42014013948. https://www.crd.york.ac.uk/prospero/display_record.php?ID=CRD42014013948	Wrong population
48	Health workforce ratios	Dejaco, C., Putrik, P., Unger, J., Aletaha, D., Bianchi, G., Bijlsma, J. W., Boonen, A., Cikes, N., Finckh, A., Gossec, L., Kvien, T. K., Madruga Dias, J., Matteson, E. L., Sivera, F., Stamm, T. A., Szekanecz, Z., Wiek, D., Zink, A., Ramiro, S., & Buttgereit, F. (2018a). EULAR 'points to consider' for the conduction of workforce requirement studies in rheumatology. RMD open, 4(2), e000780. https://doi.org/10.1136/rmdopen-2018-000780	Wrong study design
49	Health workforce ratios	Dejaco, C., Putrik, P., Unger, J., Aletaha, D., Bianchi, G., Bijlsma, J. W., Boonen, A., Cikes, N., Finckh, A., Gossec, L., Kvien, T. K., Madruga Dias, J., Matteson, E. L., Sivera, F., Stamm, T. A., Szekanecz, Z., Wiek, D., Zink, A., Ramiro, S., & Buttgereit, F. (2018b). FRI0602 EULAR 'points to consider' for the conduction of workforce requirement studies in rheumatology. Annals of the Rheumatic Diseases, 77, 825. https://doi.org/10.1136/annrheumdis-2018-eular.1774	Wrong study design
50	Health workforce ratios	Dejaco, C., Putrik, P., Unger, J., Aletaha, D., Bianchi, G., Bijlsma, J. W. J., Boonen, A., Cikes, N., Finckh, A., Gossec, L., Kvien, T., Dias, J. M., Matteson, E. L., Sivera, F., Stamm, T., Szekanecz, Z., Wiek, D., Zink, A., Ramiro, S., & Buttgereit, F. (2018). Eular 'points to consider' for the conduction of workforce requirement studies in rheumatology. Arthritis and Rheumatology, 70(Supplement 9), 1268-1269. https://doi.org/https://dx.doi.org/10.1002/art.40700	Wrong study design
51	Health workforce ratios	Di Muzio, M., Dionisi, S., Di Simone, E., Cianfrocca, C., Di Muzio, F., Fabbian, F., Barbiero, G., Tartaglini, D., & Giannetta, N. (2019). Can nurses' shift work jeopardize the patient safety? A systematic review. Eur Rev Med Pharmacol Sci, 23(10), 4507-4519. https://doi.org/10.26355/eurrev_201905_17963	Wrong intervention
52	Health workforce ratios	El-Zaemey, S., Doleman, G., Whitehead, L., & Twigg, D. (2020). The impact of different nurse staffing models on patient and nurse outcomes: a systematic review of the quantitative evidence. PROSPERO, CRD42020156717. https://www.crd.york.ac.uk/prospero/display_record.php?ID=CRD42020156717	Wrong population
53	Health workforce ratios	Fajardo Pulido, D., Arnolda, G., Giolla Easpaig, B. N., Tran, Y., Lamprell, K., Smith, J., & Braithwaite, J. (2020). Workforce planning for outpatient oncology: a systematic review of recommended staff-patient ratios. PROSPERO, CRD42020189825. https://www.crd.york.ac.uk/prospero/display_record.php?ID=CRD42020189825	Wrong population
54	Health workforce ratios	Geiger, I., Schang, L., & Sundmacher, L. (2023). Assessing needs-based supply of physicians: a criteria-led methodological review of international studies in high-resource settings. BMC Health Serv Res, 23(1), 564. https://doi.org/10.1186/s12913-023-09461-0	Wrong outcomes
55	Health workforce ratios	Gregory, W. J. (2023). E048 A review of published data on reported staffing ratios for rheumatology specialist physiotherapists in rheumatology departments. Rheumatology, 62(Supplement_2), ii148. https://doi.org/10.1093/rheumatology/kead104.297	Wrong population

56	Health workforce ratios	Griffiths, P., Recio-Saucedo, A., Dall'Ora, C., Briggs, J., Maruotti, A., Meredith, P., Smith, G. B., Ball, J., & Missed Care Study, G. (2018). The association between nurse staffing and omissions in nursing care: A systematic review. J Adv Nurs, 74(7), 1474-1487. https://doi.org/10.1111/jan.13564	Wrong intervention
57	Health workforce ratios	Halm, M. (2019). The Influence of Appropriate Staffing and Healthy Work Environments on Patient and Nurse Outcomes. Am J Crit Care, 28(2), 152-156. https://doi.org/10.4037/ajcc2019938	Wrong study design
58	Health workforce ratios	Imam, A., Obiesie, S., Aluvaala, J., Maina, J. M., Gathara, D., & English, M. (2022). Identifying gaps in global evidence for nurse staffing and patient care outcomes research in low/middle-income countries: an umbrella review. BMJ open, 12(10), e064050. https://doi.org/10.1136/bmjopen-2022-064050	Wrong study design
59	Health workforce ratios	Khalilnezhad, R., Gorji, H. A., Alaedini, F., Naeini, A. S., & Sepehri, M. M. (2020). The factors affecting the obstetricians-gynecologists workforce planning: A systematic review. Clinical Epidemiology and Global Health, 8(2), 319-328. https://doi.org/10.1016/j.cegh.2019.10.002	Wrong population
60	Health workforce ratios	Leary, A., & Punshon, G. (2019). Determining acute nurse staffing: a hermeneutic review of an evolving science. BMJ open, 9(3), e025654. https://doi.org/10.1136/bmjopen-2018-025654	Wrong study design
61	Health workforce ratios	Moyo, N., Jones, M., Kushemererwa, D., Pantha, S., Gilbert, S., Romero, L., & Gray, R. (2020). The Association between the Mental Health Nurse-to-Registered Nurse Ratio and Patient Outcomes in Psychiatric Inpatient Wards: A Systematic Review. Int J Environ Res Public Health, 17(18). https://doi.org/10.3390/ijerph17186890	Wrong outcomes
62	Health workforce ratios	Mukhopadhyay, S., Punchak, M., Rattani, A., Hung, Y. C., Dahm, J., Faruque, S., Dewan, M. C., Peeters, S., Sachdev, S., & Park, K. B. (2019). The global neurosurgical workforce: a mixed-methods assessment of density and growth. J Neurosurg, 130(4), 1142-1148. https://doi.org/10.3171/2018.10.JNS171723	Wrong study design
63	Health workforce ratios	Olds, D., Cramer, E., & Hartwell, J. (2021). The effects of nurse staffing and skill mix on patient outcomes: a systematic review of the literature and integrative model. PROSPERO, CRD42021281202. https://www.crd.york.ac.uk/prospero/display_record.php?ID=CRD42021281202	Wrong population
64	Health workforce ratios	Olley, R., Edwards, I., Avery, M., & Cooper, H. (2019). Systematic review of the evidence related to mandated nurse staffing ratios in acute hospitals. Aust Health Rev, 43(3), 288-293. https://doi.org/10.1071/AH16252	Wrong outcomes
65	Health workforce ratios	Park, S., Park, S., Lee, Y. J., Park, C. S., Jung, Y. C., & Kim, S. (2020). Nurse Staffing and Health Outcomes of Psychiatric Inpatients: A Secondary Analysis of National Health Insurance Claims Data. J Korean Acad Nurs, 50(3), 333-348. https://doi.org/10.4040/jkan.19203	Wrong study design
66	Health workforce ratios	Pastores, S. M., Kvetan, V., Coopersmith, C. M., Farmer, J. C., Sessler, C., Christman, J. W., D'Agostino, R., Diaz-Gomez, J., Gregg, S. R., Khan, R. A., Kapu, A. N., Masur, H., Mehta, G., Moore, J., Oropello, J. M., Price, K., & Academic Leaders in Critical Care Medicine Task Force of the Society of the Critical Care, M. (2019). Workforce, Workload, and Burnout Among Intensivists and Advanced Practice Providers: A Narrative Review. Crit Care Med, 47(4), 550-557. https://doi.org/10.1097/CCM.0000000000003637	Wrong study design
67	Health workforce ratios	Rae, P. J. L., Pearce, S., Greaves, P. J., Dall'Ora, C., Griffiths, P., & Endacott, R. (2021). Outcomes sensitive to critical care nurse staffing levels: A systematic review. Intensive Crit Care Nurs, 67(bg4, 9211274), 103110. https://doi.org/10.1016/j.iccn.2021.103110	Wrong population
68	Health workforce ratios	Reiter, M., & Allen, B. W. (2020). The Emergency Medicine Workforce: Shortage Resolving, Future Surplus Expected. J Emerg Med, 58(2), 198-202. https://doi.org/10.1016/j.jemermed.2020.01.004	Wrong study design
69	Health workforce ratios	Rubbo, B., Griffiths, P., Saville, C., Dall'Ora, C., Turner, L., & Ball, J. (2020). Associations between healthcare professional staffing levels and mortality in acute hospitals, a systematic review. CRD42020219869. https://www.crd.york.ac.uk/prospero/display_record.php?ID=CRD42020219869	Wrong population
70	Health workforce ratios	Safarishahrbijari, A. (2018). Workforce forecasting models: A systematic review. Journal of Forecasting, 37(7), 739-753. https://doi.org/10.1002/for.2541	Wrong population

71	Health workforce ratios	Saville, C., Griffiths, P., Ball, J., Jones, J., Dall'Ora, C., Rubbo, B., & Turner, L. (2021). Economic evaluations of alternative nurse staffing configurations: a systematic review of the literature. PROSPERO, CRD42021281202. https://www.crd.york.ac.uk/prospero/display_record.php?ID=CRD42021281202	Wrong outcomes
72	Health workforce ratios	Saville, C. E., Griffiths, P., Ball, J. E., & Monks, T. (2019). How many nurses do we need? A review and discussion of operational research techniques applied to nurse staffing. Int J Nurs Stud, 97(gs8, 0400675), 7-13. https://doi.org/10.1016/j.ijnurstu.2019.04.015	Wrong study design
73	Health workforce ratios	Saxena, S. G., & Godfrey, T. (2023). India's Opportunity to Address Human Resource Challenges in Healthcare. Cureus, 15(6), e40274. https://doi.org/10.7759/cureus.40274	Wrong study design
74	Health workforce ratios	Shin, S., Park, J. H., & Bae, S. H. (2019). Nurse staffing and hospital-acquired conditions: A systematic review. J Clin Nurs, 28(23-24), 4264-4275. https://doi.org/10.1111/jocn.15046	Wrong outcomes
75	Health workforce ratios	Stanimirovic, D., & Pribakovic Brinovec, R. (2022). Human Resource Planning in Health Care: Outlining a Basic Model and Related Complexities. Stud Health Technol Inform, 299(ck1, 9214582), 279-282. https://doi.org/10.3233/SHTI220999	Wrong study design
76	Health workforce ratios	Strobel, N., & Edmond, K. (2023). Determining health care workforce ratios for small and sick newborn care (SSNC) units: an overview of reviews. PROSPERO, CRD42023453644. https://www.crd.york.ac.uk/prospero/display_record.php?ID=CRD42023453644	Wrong study design
77	Health workforce ratios	Sutton, C., Prowse, J., McVey, L., Elshehaly, M., Neagu, D., Montague, J., Alvarado, N., Tissiman, C., O'Connell, K., Eyers, E., Faisal, M., & Randell, R. (2023). Strategic workforce planning in health and social care - an international perspective: A scoping review. Health Policy, 132, 104827. https://doi.org/10.1016/j.healthpol.2023.104827	Wrong study design
78	Health workforce ratios	Trapani, D., Murthy, S. S., Boniol, M., Booth, C., Simensen, V. C., Kasumba, M. K., Giuliani, R., Curigliano, G., & Ilbawi, A. M. (2021). Distribution of the workforce involved in cancer care: a systematic review of the literature. ESMO open, 6(6), 100292. https://doi.org/10.1016/j.esmoop.2021.100292	Wrong population
79	Health workforce ratios	Twigg, D. E., Whitehead, L., Doleman, G., & El-Zaemey, S. (2021). The impact of nurse staffing methodologies on nurse and patient outcomes: A systematic review. J Adv Nurs, 77(12), 4599-4611. https://doi.org/10.1111/jan.14909	Wrong population
80	Health workforce ratios	Villeneuve, E., Landa, P., Allen, M., Spencer, A., Prosser, S., Gibson, A., Kelsey, K., Mujica-Mota, R., Manktelow, B., Modi, N., Thornton, S., & Pitt, M. (2018). A framework to address key issues of neonatal service configuration in England: the NeoNet multimethods study. https://doi.org/https://dx.doi.org/10.3310/hsdr06350	Wrong study design
81	Health workforce ratios	Watson, S. I., Chen, Y. F., Bion, J. F., Aldridge, C. P., Girling, A., Lilford, R. J., & Hi, S. C. (2018). Protocol for the health economic evaluation of increasing the weekend specialist to patient ratio in hospitals in England. BMJ open, 8(2), e015561. https://doi.org/10.1136/bmjopen-2016-015561	Wrong study design
82	Health workforce ratios	Watts, R. D., Bowles, D. C., Ryan, E., Fisher, C., & Li, I. W. (2020). No Two Workforces Are the Same: A Systematic Review of Enumerations and Definitions of Public Health Workforces. Front Public Health, 8(101616579), 588092. https://doi.org/10.3389/fpubh.2020.588092	Wrong population
83	Health workforce ratios	Wynendaele, H., Willems, R., & Trybou, J. (2019). Systematic review: Association between the patient-nurse ratio and nurse outcomes in acute care hospitals. J Nurs Manag, 27(5), 896-917. https://doi.org/10.1111/jonm.12764	Wrong population

Appendix 12 - Figure S6. Graphical Representation of Overlap for OVErviews (GROOVE) considering chronological structural missingness

Figure A12.1 Graphical Representation of Overlap for OVErviews (GROOVE) considering chronological structural missingness – Space in SSN units

	O'Callaghan 2019
Zana-Taieb 2022	4.2%

1	=	**Total nodes (pairs of reviews)**
1	=	Slight overlap (<5%)
0	=	Moderate overlap (5% to <10%)
0	=	High overlap (10% to <15%)
0	=	Very High overlap (≥15%)

Figure A12.2 Graphical Representation of Overlap for OVErviews (GROOVE) considering chronological structural missingness – Health workforce ratios in SSN units

	Bosak 2023	Lee 2024	Lopes 2021	Sharma 2020
Lee 2024	12.8%			
Lopes 2021	7.1%	17.3%		
Sharma 2020	0.0%	0.0%	0.0%	
Rafiei 2019	0.0%	0.0%	2.6%	0.0%

10	=	**Total nodes (pairs of reviews)**
7	=	Slight overlap (<5%)
1	=	Moderate overlap (5% to <10%)
1	=	High overlap (10% to <15%)
1	=	Very High overlap (≥15%)

Appendix 13 - Figure S7. ROBIS assessment of the included studies

Figure A121.1 ROBIS assessment of Space in NICUS studies

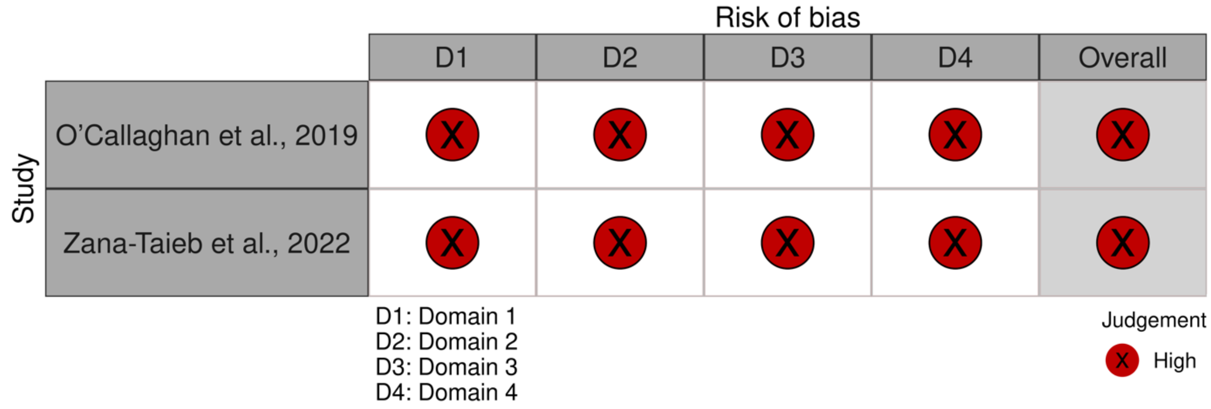

Figure A11.2 ROBIS assessment of Health workforce ratios studies

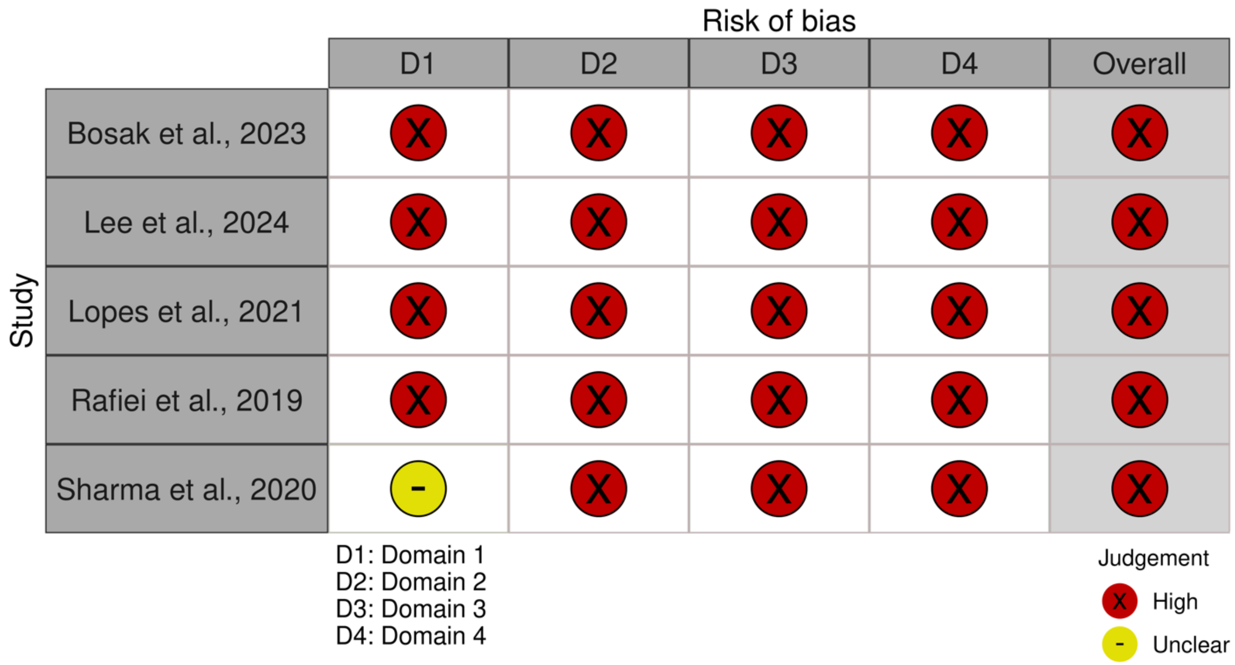

Figure A11.3 ROBIS assessment of Time to Travel studies

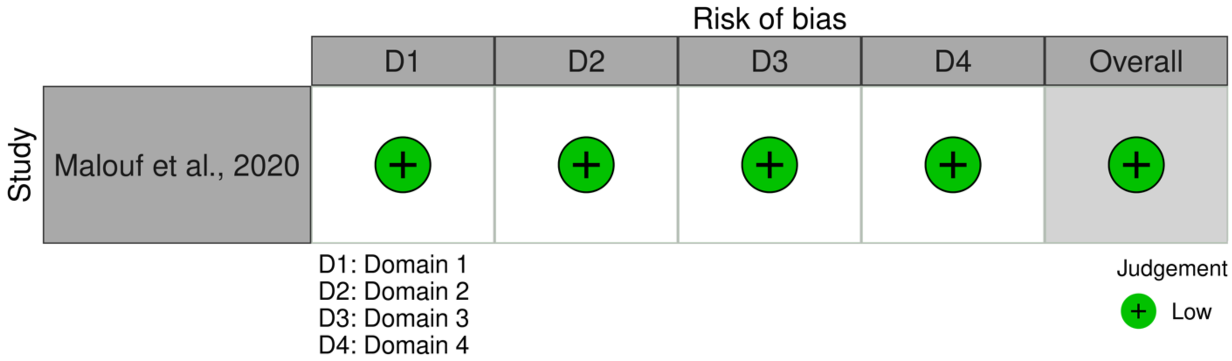

Appendix 14 – Table S7. Methodological assessment of included studies

Domain	Review name	Tool used by study to assess risk of bias	Tool used by study to assess certainty of evidence	Tool used by study to develop recommendations	Overlap of primary studies calculated by GROOVE tool *	Risk of bias assessment (ROBIS)
Space in NICUs	O'Callaghan 2019 [19]	NR	NR	NR	Slight overlap (4.17%)	High
Space in NICUs	Zana-Taieb 2022 [20]	NR	NR	NR		High
Health workforce ratios	Bosak 2023 [21]	NR	NR	NR	Slight overlap (3.29%)	High
Health workforce ratios	Lee 2024 [22]	NR	NR	NR		High
Health workforce ratios	Lopes 2021 [23]	NR	NR	NR		High
Health workforce ratios	Rafiei 2019 [24]	NR	NR	NR		High
Health workforce ratios	Sharma 2020 [25]	NR	NR	NR		High
Travel time	Malouf 2020 [26]	Newcastle-Ottawa scale (NOS)	NR	NR	NA	Low

NICU-neonatal intensive care unit; NA-not applicable; NR-not reported

* **A**djusted for chronological missingness [15,16]
